# Familial hemophagocytic lymphohistiocytosis hepatitis is mediated by IFN-γ in a predominantly hepatic-intrinsic manner

**DOI:** 10.1371/journal.pone.0269553

**Published:** 2022-06-07

**Authors:** Tamir Diamond, Thomas N. Burn, Mailyn A. Nishiguchi, Danielle Minichino, Julie Chase, Niansheng Chu, Portia A. Kreiger, Edward M. Behrens

**Affiliations:** 1 Division of Gastroenterology Hepatology and Nutrition, Children’s Hospital of Philadelphia, Philadelphia, PA, United States of America; 2 Perlman School of Medicine at the University of Pennsylvania, Philadelphia, PA, United States of America; 3 Division of Rheumatology, Children’s Hospital of Philadelphia, Philadelphia, PA, United States of America; 4 Department of Pathology, Children’s Hospital of Philadelphia, Philadelphia, PA, United States of America; Instituto Nacional de Ciencias Medicas y Nutricion Salvador Zubiran, MEXICO

## Abstract

Interferon gamma (IFN-γ) is the main cytokine driving organ dysfunction in Familial Hemophagocytic Lymphohistiocytosis (FHL). Blockade of IFN-γ pathway ameliorates FHL hepatitis, both in animal models and in humans with FHL. Hepatocytes are known to express IFN-γ receptor (IFN-γ-R). However, whether IFN-γ induced hepatitis in FHL is a lymphocyte or liver intrinsic response to the cytokine has yet to be elucidated. Using a *IFNgR*^*−/−*^ bone marrow chimeric model, this study showed that non-hematopoietic IFN-γ response is critical for development of FHL hepatitis in LCMV-infected *Prf1*^*−/−*^ mice. Lack of hepatic IFN-γ responsiveness results in reduced hepatitis as measured by hepatomegaly, alanine aminotransferase (ALT) levels and abrogated histologic endothelial inflammation. In addition, IFN-γ non-hematopoietic response was critical in activation of lymphocytes by soluble interleukin 2 receptor (sIL-2r) and recruitment of CD8+ effector T lymphocytes (CD8+ CD44^hi^ CD62L^lo^) (Teff) and inflammatory monocytes. Lastly, non-hematopoietic IFN-γ response results in increased hepatic transcription of type 1 immune response and oxidative stress response pathways, while decreasing transcription of genes involved in extracellular matrix (ECM) production. In summary, these findings demonstrate that there is a hepatic transcriptional response to IFN-γ, likely critical in the pathogenesis of FHL hepatitis and hepatic specific responses could be a therapeutic target in this disorder.

## 1. Introduction

Cytokine Storm Syndromes (CSS) are a group of disorders of various etiologies that culminate in a common final pathway of systemic inflammation affecting multiple organs and driving high rates of mortality [1]. Acute liver injury resulting in rapid decompensation in function is a common manifestation of many CSS, including familial hemophagocytic lymphohistiocytosis (FHL) and macrophage activation syndrome (MAS). Hyperactivation of effector CD8+ T-cells producing abundant interferon gamma (IFN-γ) are the main immunopathological drivers in these disorders clinically and in experimental models [2–5].

FHL type 2 (FHL2) is caused by mutations in the perforin gene (*Prf1*), which results in dysfunctional cytolysis by CD8+ T-cells causing persistent antigen exposure. Consequently, CD8+ T-cell proliferation and activation, with excessive production of cytokines such as IFN-γ, results in multisystemic organ dysfunction with high morbidity and mortality [6]. A murine model of FHL2 (*Prf1*^*-/-*^) develops a phenotype similar to human disease and has been extensively used to elucidate cytokine storm syndromes in general and FHL specifically [2]. The work in this model demonstrating depletion of CD8+ T cells and blockade of IFN-γ ameliorating disease provided the pre-clinical basis for development of a novel treatment for blocking this cytokine in human disease (empalumab) [7–10].

The liver is an organ with a unique immunological milieu. Its role as a filter barrier to the outside environment for metabolically essential nutrients as well as pathogens and harmful toxins, necessitate its complex interaction with the immune system [11]. Hepatocytes have an IFN-γ receptor (IFNγ-R) that induces an antiviral, anti-tumor and tissue regenerative response, including upregulation of anti-viral defense, cell cycle arrest and apoptosis pathways [12–14]. *In vitro* experiments and *in vivo* models of CSS describe the role of IFN-γ in hepatocyte injury [4, 5, 15–17]. Moreover, recent publications from our group showed that in the FHL2 (*Prf1*^*-/-*^) model, canonical signals of tissue injury may contribute to FHL pathophysiology in addition to IFN-γ leukocyte response in the liver [18, 19]. However, despite our knowledge that CD8+ T cell production of IFN-γ plays a significant role in pathogenesis of FHL, there are no studies to date that assess the targets of IFN-γ, either acting directly on liver tissue, or on immune cells, to initiate or promote injury.

In the current study, we sought to determine if IFN-γ-mediated hepatic injury in the murine model of FHL is caused by direct effect of IFN-γ on the liver, or by IFN-γ acting on lymphocytes that in turn mediate injury. We used bone marrow (BM) chimeras to assess how non-hematopoietic response to IFN-γ signaling in FHL model may result in hepatitis by using lethal irradiation of hosts and injection of BM cells from donors that were either IFNγ-R deficient or sufficient. Our observations establish that lack of IFN-γ response in the liver as a non-hematopoietic organ is hepato-protective independent of leukocyte response to IFN-γ. In addition, we found that non-hematopoietic IFN-γ response is necessary for recruitment of inflammatory monocytes and CD8+ effector T lymphocytes (CD8+ CD44^hi^ CD62L^lo^) (Teff). Lastly, transcriptomic analysis of hepatic IFN-γ response showed alteration in cellular metabolic oxidative stress response and production of ECM in addition to inflammatory pathways.

## 2. Materials and methods

### 2.1. Mice

C57BL/6 (wild-type), perforin-deficient (C57BL/6-Prf1tm1Sdz/J, *Prf1*^*−/−*^), IFN-γ-R–deficient (B6.129S7-Ifngr1tm1Agt/J, *IFNgR*^−/−^), mice were purchased from The Jackson Laboratory and bred in our facility. *Prf1*^−/−^ mice were cross-bred with *IFNgR*^−/−^ mice to create *Prf1*^−/−^ *IFNgR*^−/−^. Euthanasia was achieved via CO2 chamber and cardiac puncture. Anesthesia performed using 3% Isoflurane with 2% oxygen delivered in anesthesia chamber. All animal studies were performed with approval from The Children’s Hospital of Philadelphia Institutional Animal Care and Use Committee.

### 2.2. Generation of bone marrow chimeras

BM chimeras were created to assess hepatocyte response to IFN-γ signaling in FHL model. All mice were deficient in *Prf1 (Prf1*^*−/−*^) and either deficient or sufficient in *IFNgR* expression. Mice 6-11-week-old Perforin-deficient (*Prf1*^*−/−*^ *IFNgR*^*+/+*^) and Perforin-IFN-γ-R–deficient (*Prf1*^*-/-*^*IFNgR*^*−/−*^) hosts were lethally irradiated (950 Rad on an X-RAD irradiator). BM was isolated from donor strains (*Prf1*^*−/−*^ *IFNgR*^*+/+*^ or *Prf1*^*-/-*^*IFNgR*^*−/−*^) and 3-5x10^6^ BM cells were injected intravenously to host 6 hours after irradiation. We generated all pairwise combinations of genotype in marrow or host resulting in *Prf1*^-/-^ mice that were selectively deficient in IFN-γ-R in the bone marrow, non-hematopoietic tissues, both of these compartments, or neither compartment. All chimeras were normal in appearance and behavior prior to any infections.

### 2.3. Induction of FHL and acute hepatitis

8–14 weeks after creation of BM chimeras to allow for marrow reconstitution, chimeric mice were infected intraperitoneally with 2X10^5^ plaque-forming units of Lymphocytic Choriomeningitis Virus (LCMV) (Armstrong strain). Mice were sacrificed 8 days after infection when features of FHL become apparent. Mice were euthanized earlier if developed significant morbidity or weight loss (e.g >20%) [18–20].

Alanine aminotransferase (ALT) and aspartate aminotransferase (AST) were measured from serum using a Roche Cobas c311 clinical chemistry analyzer. Complete blood count with differential (CBC) was analyzed using Sysmex XT-2000i hematology analyzer. Serum IFN-γ and soluble interleukin-2 receptor (sIL-2r) were measured by enzyme-linked immunosorbent assay (ELISA) using BD Biosciences and R&D Systems commercial kits respectively according to protocol.

### 2.4. Histologic analysis

Unperfused livers were fixed for 24 hours in 4% paraformaldehyde and either stained with hematoxylin and eosin (H&E) or embedded in paraffin. Slides were read with grading of lobular inflammatory score, necrosis, steatosis, portal inflammation and endothelial injury score by pediatric pathologist blinded to treatment protocols (PAK). The established grading criteria of endothelial injury were previously published [21]. Images were acquired on an Eclipse 90i microscope (Nikon, Melville, NY) using NIE-ELEMENTS software.

### 2.5. Flow-cytometric analysis

Intrahepatic leukocytes were isolated using a Percoll (GE Healthcare Life Sciences) density gradient centrifugation. 2/3 of each liver was disrupted using a 70-micron filter. The cell pellet was resuspended in 30% Percoll, layered over 70% Percoll, and centrifuged. The interface formed contained the intrahepatic leukocytes and stained with LIVE/DEAD fixable viability dye (Life Technologies) and CD4, CD8α, NK1.1, B220, Ly6C, Ly6G, CD11b, CD44, CD62L, CXCR3 and CD90.2 (BD, Pharmingen, eBioscience and BioLegend). Splenocytes were stained with LIVE/DEAD fixable viability dye and B220, CD44, CD4, CD90.2, CD62L, CD8α (S1 Table). All samples were acquired on a MACSQuant flow cytometer (Miltenyi Biotec) and analyzed using FlowJo software version 10.6 (Tree Star). (See S1 and S2 Figs for gating strategy.)

### 2.6. Messenger RNA sequencing (mRNA-seq) data analysis and visualization

Fresh mouse liver segments were stored in RNAlater® solution (Thermo Fisher Scientific) at -20°C and thawed for RNA extraction at later date. RNA extraction was performed in livers of 4 mice from the BM *IFNgR+/+* and host *IFNgR -/-* group and 4 mice from the BM *IFNgR*+/+ and host *IFNgR*+/+ group using the Qiagen RNeasy Mini Kit protocol from approximately 30mg of homogenized tissue according to manufacturer instructions. Prepared RNA samples and libraries were assessed for quality and quantified using an Agilent Tapestation 4200 and Qubit 3, respectively. Libraries were prepared using the Illumina Truseq stranded mRNA library prep (Illumina). Samples were sequenced on an Illumina NextSeq 500 to produce 65 bp single end reads with a mean sequencing depth of 4 million reads per sample.

Raw reads were mapped to the mouse reference transcriptome (Ensembl, Mus musculus version 101) using Kallisto version 0.46.0 [22]. All subsequent analyses were carried out using the statistical computing environment, R (version 4.0.0) in RStudio (version 1.1.453) and Bioconductor (version 3.11). The TxImport package was used to summarize transcript quantification data to genes [23]. Data was normalized using the TMM method in EdgeR and filtered for genes with < 1 count per million (CPM) in 4 samples (the number of replicates per genotype) [24]. Processed data were variance-stabilized using the VOOM function in limma [25]. After correcting for multiple testing using Benjamini-Hochberg, linear modeling using limma (FDR ≤ 0.05; absolute logFC ≥ 1), was performed to identify differentially expressed genes. Gene ontology (GO) analysis was performed using Database for Annotation, Visualization and integrated Discovery (DAVID) (https://david.ncifcrf.gov/) Raw sequence data is available on the Gene Expression Omnibus (GEO, accession #GSE168086).

### 2.7. Quantitative real-time polymerase chain reaction

RNA was isolated from snap frozen livers in OCT using TRIzol LS (Thermo Fisher Scientific) with RNAeasy Mini Kit (Qiagen). Isolated RNA was converted to complementary DNA using the Superscript III First-Strand Synthesis System (Life Technologies) and subjected to quantitative real-time polymerase chain reaction using QuantiTect primers for *Cxcl9*, *Igtp1*, *Irgm1* and *Stat1* (Qiagen) and Power SYBR Green master mix (Life Technologies). Results were normalized to β-actin (*Actb)* using ΔΔCT method.

### 2.8. Quantitative Glutathione S-Transferase activity

Fresh mouse liver tissue was extracted, sectioned and weighed so all samples were 50mg. Tissue was rinsed in phosphate buffered saline (PBS) to remove blood. Tissue was the homogenizer in 250 μL of cold 100mM potassium phosphate, pH 7 with 2mM EDTA. All samples were subsequently frozen in -80° C to lyse cells. After freezing and thaw cycle, specimens were centrifuged at 10,000x g for 15 minutes at 4° C. Supernatant from samples was removed and assayed using Invitrogen™ Glutathione S-Transferase (GST) Fluorescent Activity Kit (Thermo Fischer Scientific) according to instructions.

### 2.9. Statistical analysis

All data were analyzed in GraphPad Prism 8 using statistical tests indicated in the figure legends and results section. Unless otherwise specified, *P*-values are represented by number or symbols (e.g *P<0.05, ** *P*<0.01, ***P<0.001).

### 2.10. Data sharing

The raw data supporting the conclusion in this manuscript will be made available by the authors, without undue reservation, to any qualified researcher.

## 3. Results

### 3.1. Bone marrow IFN-γ signaling results in pronounced features of FHL

**Fig 1:**
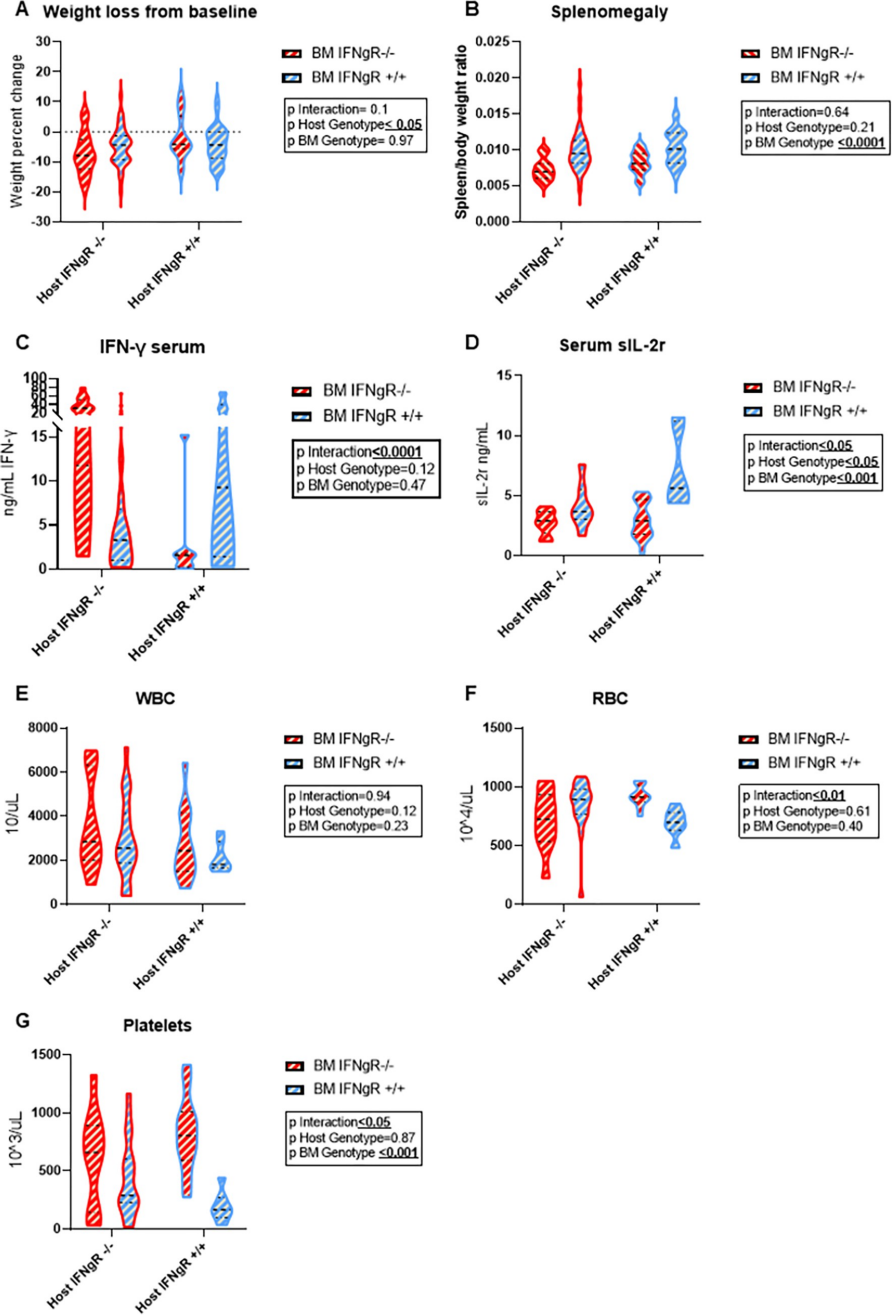
Systemic FHL characteristics are primarily dependent on IFN-γ response in the BM. Graphs illustrating characteristics of FHL: (A) Weight loss on day 8 compared to baseline (n≥15mice per group), (B) splenomegaly ratio = spleen weight/total weight (n≥15 mice per group), (C) serum IFN-γ and (D) sIL-2r (n≥6 mice per group) and (E) white blood cell (WBC), (F) red blood cell (RBC) and (G) platelet counts (n≥7 mice per group) were analyzed for BM and non-hematopoietic IFN- γ-R effects as well as interactions between these compartments using 2 way ANOVA. Blue represents *IFNgR+/+* and red *IFNgR-/-* in BM (zebra filling) and non-hematopoietic (violin plot border). P-values for each effect are denoted in box embedded in the graphs; medians and quartiles are depicted in the dashed and dotted lines respectively.

Consistent with our previous published results, all groups developed certain characteristics of FHL independent of IFN-γ-R response in either the host or BM, including weight loss (Fig 1A), splenomegaly (Fig 1B), elevated serum IFN-γ, sIL-2r (Fig 1C and 1D), lower end of normal range of leukocytes (WBC), red blood cells (RBC) and thrombocytopenia (Fig 1E–1G) [19, 26]. There was no significant difference in day 8 mortality between groups with all having less than 9% mortality. Serum IFN-γ levels were highest in completely receptor deficient (*IFNgR*^*-/-*^ BM in host *IFNgR*^*-/-*^) group, compared to the BM deficient only (*IFNgR*^*-/-*^ BM in host *IFNgR*^*+/+*^) and host deficient only (*IFNg*^*+/+*^ BM in host *IFNgR*^*-/-*^) groups, with an intermediate level in completely sufficient (*IFNgR*^*+/+*^ BM in host *IFNgR*^*+/+*^) mice (Fig 1C). This supports previous data showing serum IFN-γ levels are not only modified by Teff production via a positive IFN-γ feedback loop, but also consumption by IFN-γ-R, in both leucocytes and non-immune tissue [19]. In addition, sIL-2r serum levels were significantly elevated in all groups compared to uninfected WT (mean 439.8pg/mL range 159.24–645.0.5, data not shown) and previously reported LCMV infected WT [19]. sIL-2r levels were dependent on both BM and host IFN-γ-R response in a synergistic interaction (Fig 1D). This suggests that IFN-γ effect on non-immune organs contributes to lymphocyte activation in addition to a lymphocyte intrinsic response. Notably, splenomegaly was entirely driven by BM IFN-γ-R response (Fig 1B), while host IFN-γ-R signaling reduced the degree of weight loss (Fig 1A). The degree of thrombocytopenia was driven mainly by BM IFN-γ -responsiveness. However, when there was non-hematopoietic signaling of IFN-γ (*IFNgR*^*-/-*^ BM in *IFNgR*^*+/+*^ host), thrombocytopenia was milder compared to whole body *IFNgR*^*-/-*^ group (Fig 1G).

### 3.2. Non-hematopoietic IFN-γ response produces murine FHL hepatitis and endothelial injury in a cell intrinsic manner

**Fig 2:**
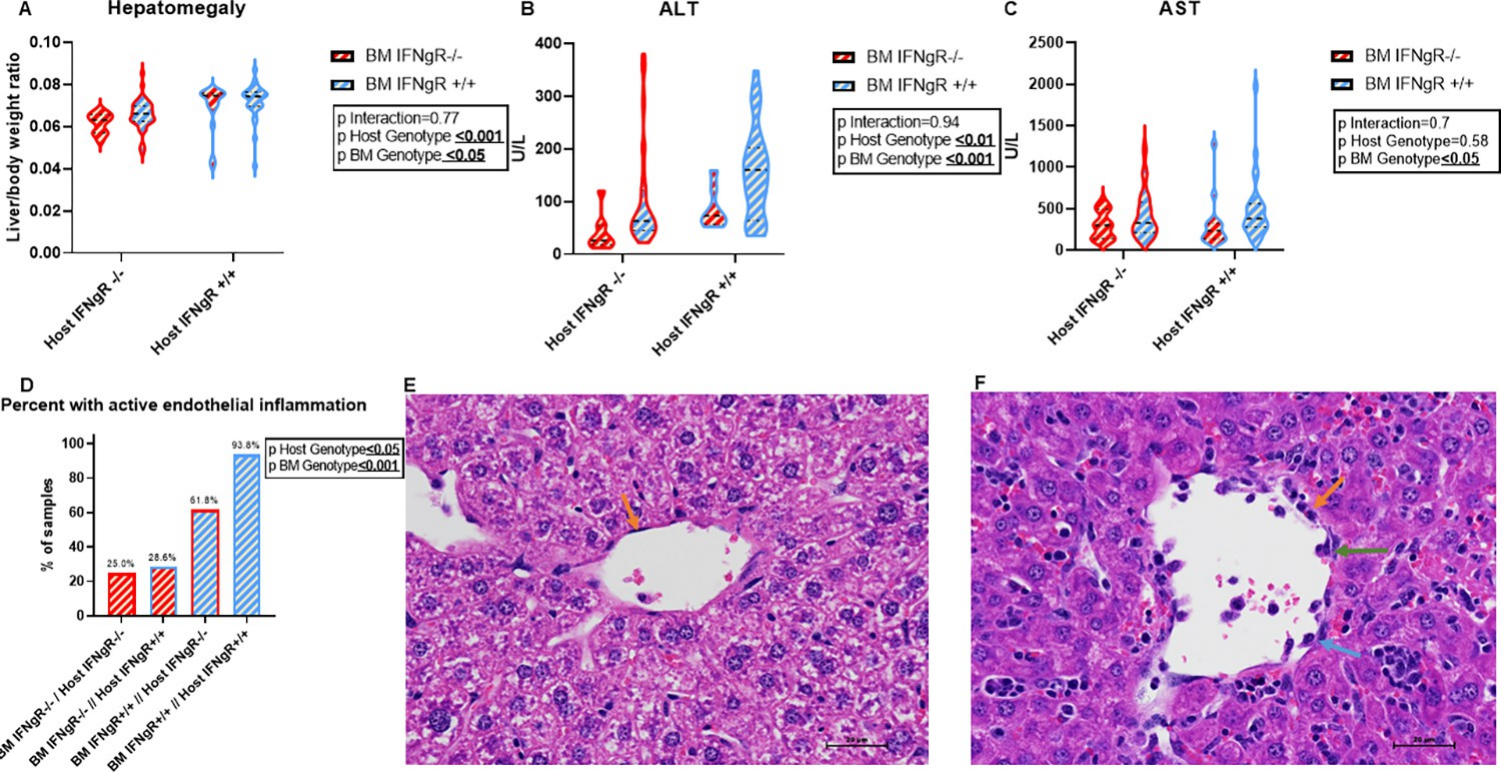
Hepatic IFN-γ receptor response results in worsening liver injury in FHL. Degree of liver injury was assessed quantitatively by (A) Hepatomegaly ratio = liver weight/total weight, (B)ALT and (C) AST (n ≥ 15 mice per group) were analyzed for BM and non-hematopoietic IFN- γ-R effects as well as interactions between these compartments using 2 way ANOVA. Blue represents *IFNgR+/+* and red *IFNgR-/-* in BM (zebra filling) and non-hematopoietic (violin plot border). P-values for each effect are denoted in box embedded in the graphs; medians and quartiles are depicted in the dashed and dotted lines respectively. Histological analysis of liver injury (D) quantitatively, percent of samples with active endothelial inflammation (score ≥1), using multiple logistic regression with p values denoted in the box embedded in the graph, N≥7 mice per group. Qualitatively, H&E sections at 400x total magnification. (E) Normal central vein with no evidence of endothelial cell activation in *IFNgR-/-* liver. Endothelial cells appear thin, elongated, and attenuated along the vessel wall and contain thin elongated bland nuclei (orange arrow). In contrast, in (F) *IFNgR +/+* liver slide showing abnormal central vein with evidence of endothelial cell activation. Endothelial cells appear plump with rounded nuclei (orange arrow). Some endothelial cells appear to bulge (green arrow) and even potentially slough (blue arrow) into the vascular lumen.

We investigated the contribution of IFN-γ-R signaling in the hepatic and BM derived leukocyte response in hepatitis using a 2-way ANOVA model. To determine variance in hepatic IFN-γ-R response was from BM derived leukocytes or liver intrinsic signaling we preformed quantitative PCR for liver extracted mRNA of 4 canonical downstream genes- chemokine ligand 9 (*Cxcl9)*, interferon gamma induced GTPase (*Igtp1)*, immunity-related GTPase family M member 1 (*Irgm1)* and signal transducer and activator of transcription 1 (*Stat1)*. BM IFN-γ-R response did not contribute significantly to hepatic transcription to any of the 4 interferon-γ-inducible genes (S1 Fig). This suggested there was a transcriptional hepatic interferon-γ response that was independent of BM response. As expected, there was a significant contribution of BM IFN-γ-R response to hepatocyte injury. However, the non-hematopoietic IFN-γ-R response worsened liver injury, independent of BM driven injury as measured by hepatomegaly and ALT (Fig 2A and 2B). Interestingly, AST, a less specific marker for hepatocellular injury, did not show a significant difference in non-hematopoietic IFN-γ-R mediated response (Fig 2C). Histologically, livers analyzed from hosts that were *IFNgR*^*-/-*^ (Fig 2E) had a significantly lower percent of endothelial inflammatory activity compared to *IFNgR*^*+/+*^ livers (Fig 2F). This host effect was independent of BM IFN-γ-R response when analyzed by multiple logistic regression analysis (Fig 2D–2F).

### 3.3. Non-hematopoietic IFN-γ response contributes to CD8+ T effector cell (CD8+ CD44^hi^ CD62L^lo^) and inflammatory monocyte predominance in hepatic inflammation

After observing the non-hematopoietic cell contribution of IFN-γ-R response to liver injury, we evaluated the hepatic inflammatory milieu as compared to peripheral blood and spleen (S2 & S3 Figs for gating strategy). There was no significant difference in either BM or host IFN-γ-R response in peripheral blood monocytes, lymphocytes and neutrophils, although the host *IFNgR*^*-/-*^ groups showed a trend of neutrophilia. (S4A–S4C Fig). Splenic B-cell and naïve CD8+ T-cells (CD8+ CD44^lo^ CD62L^hi^) cells were significantly higher when lymphocytes were unresponsive to IFN-γ and the non-hematopoietic IFN-γ response was intact (S4D and S4E Fig). This interaction was not observed in the splenic Teff cell population (S4F Fig). This suggests mobilization from lymphoid tissue of B-cells and naïve CD8 T-cells to periphery is controlled by a complex interplay of lymphocyte intrinsic and non-immune organ response to IFN-γ.

**Fig 3:**
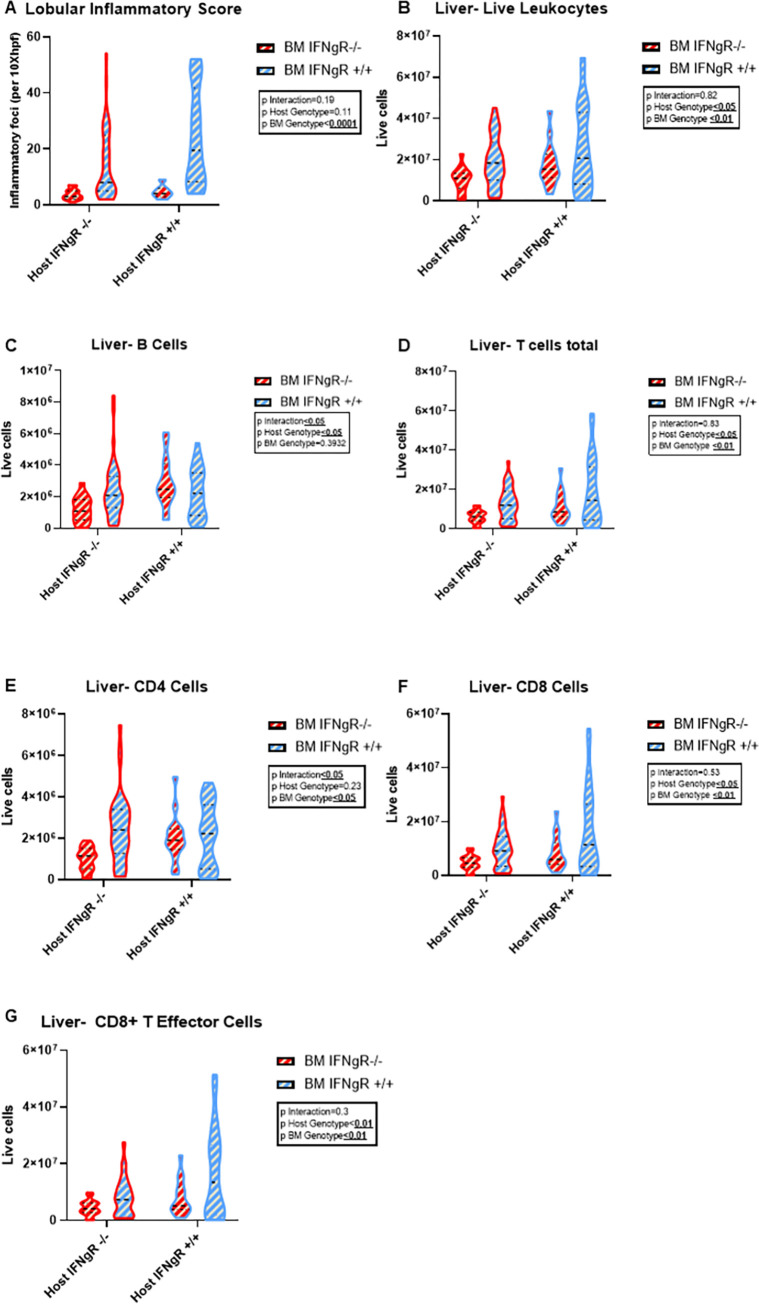
Non-hematopoietic IFN-γ receptor response is responsible for hepatic CTL recruitment, primarily T effector cells (CD8+ CD44hi CD62Llo), in an intrinsic manner. Histological analysis of hepatic inflammation: (A) inflammatory foci were quantified on H&E slides using Lobular Inflammatory Score per 10Xhpf, n ≥ 7 mice per group. Flow cytometry analysis of (B) live leukocytes, (C) B cells (B220+, CD90.2-) and (D) T cells (B220-, CD90.2+). T cells subpopulation quantified; (E) CD4+ T-helper cells, (F) CD8+ and (G) specifically T Effector Cells (CD8+ CD44hi CD62Llo) (n ≥ 14 mice per group). All data were analyzed for BM and non-hematopoietic IFN- γ-R effects as well as interactions between these compartments using 2 way ANOVA. Blue represents *IFNgR+/+* and red *IFNgR-/-* in BM (zebra filling) and non-hematopoietic (violin plot border). P-values for each effect are denoted in box embedded in the graphs; medians and quartiles are depicted in the dashed and dotted lines respectively.

Histologic assessment of hepatic inflammation revealed a lobular inflammatory score demonstrating inflammatory recruitment is mainly driven by BM IFN-γ-R response with a trend showing non-hematopoietic contribution that was not statistically significant (Fig 3A). However, a more quantitative analysis using flow cytometry of intrahepatic leukocytes showed an independent contribution of hepatic IFN-γ-R response to leukocyte recruitment (Fig 3B). Non-hematopoietic IFN-γ-R signaling was critical for hepatic lymphocyte recruitment of both B Cells (Fig 3C) and T-cells for which BM and hepatic IFN-γ-R signaling contributed independently (Fig 3D). When evaluating the different T-cell sub-populations, recruitment of hepatic CD8+ T-cells, particularly Teff (CD8+ CD44^hi^ CD62L^lo^) cells, were dependent on non-hematopoietic IFN-γ-R response, independently of the BM response (Fig 3E–3G).

**Fig 4:**
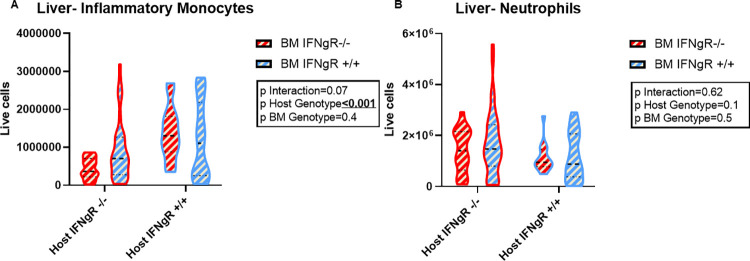
Inflammatory monocyte recruitment is determined by hepatic IFN-γ receptor. Quantitative assessment of myeloid cells (Live, CD90.2-, B220-) by flow cytometry. (A) Inflammatory Monocytes (Ly6G-, CD11b+, Ly6C+) and (B) Neutrophils (Ly6G+,CD11b+) infiltrates in liver (n ≥ 14 mice per group). All data were analyzed for BM and non-hematopoietic IFN- γ-R effects as well as interactions between these compartments using 2-way ANOVA. Blue represents *IFNgR+/+* and red *IFNgR-/-* in BM (zebra filling) and non-hematopoietic (violin plot boarder). P-values for each effect are denoted in box embedded in the graphs; medians and quartiles are depicted in the dashed and dotted lines respectively.

Liver myeloid infiltration was also under the control of non-hematopoietic IFN-γ-R. Inflammatory monocytes (Ly6C+, Ly6G-, CD11b+) were significantly elevated in livers that were IFN-γ-R responsive (Fig 4A). We previously described the regulation of neutrophil survival in FHL being mediated by IFN-γ in a neutrophil-intrinsic manner. The IFN-γ-R neutrophil response results in increased apoptosis rather than decrease in BM production [19]. In this current study, we observed an increase in hepatic neutrophil recruitment when liver IFN-γ-R signaling was absent, but this was not statistically significant (Fig 4B). These findings suggest that IFN-γ regulates inflammatory monocyte tissue infiltration predominantly by the non-hematopoietic response to the cytokine directly but that neutrophil responses are largely cell-intrinsic.

### 3.4. Non-hematopoietic IFN-γ response increases hepatic cellular stress pathways and decreases extracellular matrix production in FHL

To determine the downstream effects of non-hematopoietic intrinsic IFN-γ response on the transcriptional level, we performed messenger RNA sequencing (mRNAseq) from IFN-γ-R deficient (*IFNgR*^*-/-*^) and sufficient (*IFNgR*^*+/+*^) livers, both reconstituted with IFN-γ-R-sufficient bone marrow (*IFNgR*^*+/+*^ BM*)* in our chimeric FHL model. As we described above, the number of intrahepatic leukocytes was modestly affected by hepatic IFN-γ response (Fig 3B). However, leukocytes still make up a minority of the cells in these livers, meaning that the bulk of the mRNA extracted will be of liver origin and therefore unlikely to contribute to transcriptional differences between groups. Consistent with the notion that hepatic leukocyte variance between groups was minimal, there was no significant difference in *PTPRC* (which encodes the leukocyte marker CD45) transcription between groups (data not shown but accessible at GEO #GSE168086). Additionally, as described above in section 3.2 BM IFN-γ-R response did not contribute significantly to hepatic transcription of downstream interferon-inducible genes of the IFN-γ pathway (S1 Fig).

**Fig 5:**
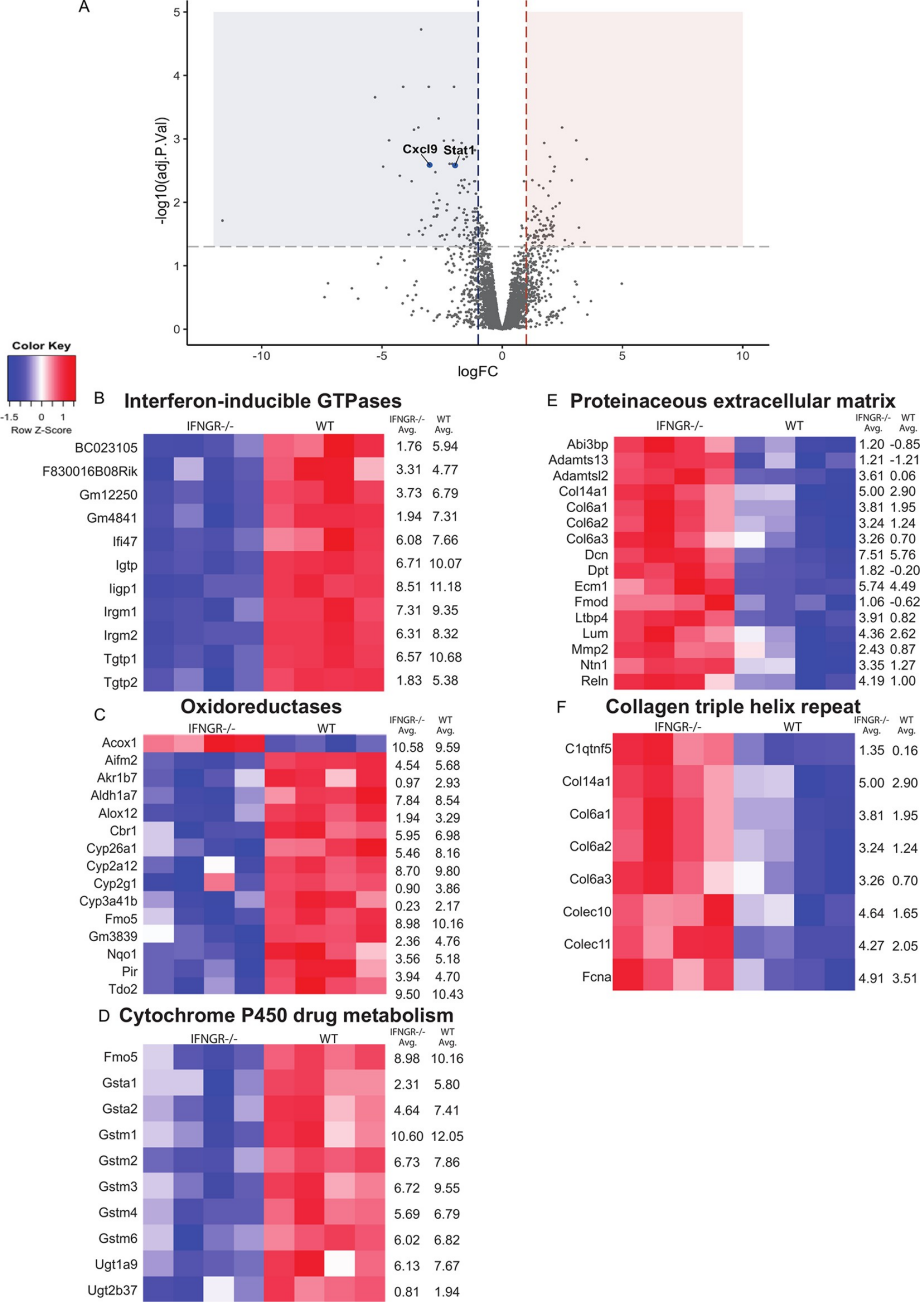
Non-hematopoietic IFN-γ response increases cellular stress response and reduces ECM production in the liver. Mice with *IFNgR+/+* BM and either *IFNgR+/+* or *IFNgR-/-* livers (n = 4 per group) were sacrificed 8 days after LCMV infection to induce FHL hepatitis and livers were harvested for mRNAseq analysis. (A) Volcano plot comparing genes that transcripts were decreased (shaded in blue) and increased (shaded in red) in *IFNgR-/-* livers when compared to *IFNgR+/+* livers as reference. X axis notes the log fold change (logFC) using *IFNgR+/+* livers as reference and Y axis notes the log adjusted p value [-log10(adj.p.val)]. Heatmaps showing relative expression from the individual genes from the pathways (B-D) upregulated and (E-F) downregulated by the presence of IFNγ- R according to GO analysis using DAVID. Genes are printed on left of heatmap and reads Per Kilobase of transcript, per Million mapped reads (RPKM) averages are provided to right of heatmap.

Comparing *IFNgR*^*+/+*^ to *IFNgR*^*-/-*^ livers, there was a higher number of transcripts of *Stat1* and *Cxcl9* in the *IFNgR*^*+/+*^ livers (Fig 5A), which was confirmed using quantitative real-time polymerase chain reaction (S1 Fig). This is correlated with a non-hematopoietic IFN-γ-R dependent decrease in chemokine receptor 3 (CXCR3)+ surface expression in liver CD8+ Teff and CD4+ T cells, an event well described in response to CXCL9 (S5–S7 Figs) [27]. Furthermore, infiltration of CXCR3+ inflammatory monocytes is decreased in IFNgR^-/-^ livers, particularly when IFN-γ-R is absent in the bone marrow, suggesting a non-hematopoietic intrinsic role for IFN-γ induced CXCL9 in monocyte recruitment (S5C and S5D Fig). In addition, transcripts of Mixed lineage kinase domain like pseudokinase gene (*MLKL)*, which is a known mediator of hepatocyte death induced by IFN-γ, were increased in *IFNgR*^*+/+*^ livers, indicative of direct IFN-γ mediated hepatocyte injury (Fig 5A and S2 Table) [28, 29]. This result confirmed a differential IFN-γ response in livers that were receptor sufficient (*IFNgR*^*+/+*^) compared to deficient (*IFNgR*^*-/-*^). Unsurprisingly, analysis of differentially expressed genes via GO analysis using the DAVID toolset, found a transcriptional enrichment in both innate and type 1 adaptive immune response in *IFNgR*^*+/+*^ livers compared to *IFNgR*^*-/-*^ (Table 1, Fig 5B and S2 Table).

**Table 1:** Gene pathways enriched in IFNγ-R+/+ livers.

Gene Set Origin	Pathway Function	Number of genes in cluster (% of total)	Benjamini score (adj. p value)
INTERPRO	Interferon-inducible GTPase	11 (7.3)	2.9E-15
GOTERM_BP_DIRECT	Cellular Response to interferon-beta	13 (8.6)	4.1E-14
GOTERM_MF_DIRECT	GTP binding	22 (14.6)	2E-10
KEGG_PATHWAY	Drug metabolism—cytochrome P450	10 (6.6)	2.6E-7
UP_KEYWORDS	Oxidoreductase	15 (9.9)	4.6E-3
INTERPRO	Glutathione S-transferase, C-terminal	7 (4.6)	3.2E-6
INTERPRO	MHC class I-like antigen recognition	9 (6)	4.5E-7
KEGG_PATHWAY	Antigen processing and presentation	10 (6.6)	1.1E-6

Interestingly, there was an enrichment in pathways associated with gene transcription of cytochrome P-450 (CYP450) drug metabolism by GST activity as well as oxidoreductases in *IFNgR*^*+/+*^ livers compared to *IFNgR*^*-/-*^. These genes included those involved in glutathione metabolism as well as certain CYP450 isoforms (Cyp26a1, Cyp2a12, Cyp2g1, Cyp3a41b) (Table 1 and Fig 5C and 5D and S2 Table). We evaluated if transcriptional enrichment correlates to hepatic GST enzymatic activity using fluorescent activity assay. GST activity was entirely dependent on hepatic IFN-γ-R response, which correlated with decrease enzymatic activity in livers sufficient for the receptor (S8 Fig) This is consistent with a hepatic response to IFN-γ that results in decreased GST activity and a compensatory transcriptional increase in GST mRNA. In addition to these differences, the main pathways that were transcriptionally enriched in the *IFNgR*^*-/-*^ livers compared to *IFNgR*^*+/+*^ were those involved in collagen synthesis and extra cellular matrix (ECM) production (Table 2, Fig 5E and 5F and S3 Table). These results indicate that IFN-γ mediated ECM remodeling disruption is an intrinsic transcriptional element of hepatic pathology in FHL and correlates to the parenchymal collapse described histologically in FHL hepatitis in humans [30].

**Table 2:** Gene pathways enriched in IFNγ-R-/- livers.

Gene Set Origin	Pathway Function	Number of genes in cluster (% of total	Benjamini score (adj. p value)
UP_KEYWORDS	Disulfide bond	46 (57.5)	1.8E-17
GOTERM_CC_DIRECT	Proteinaceous extracellular matrix	16 (20)	5.9E-11
INTERPRO	Collagen triple helix repeat	8 (10)	2.7E-6
UP_KEYWORDS	Secreted mediators	36 (45)	1.8E-17

## 4. Discussion

This study reveals that non-hematopoietic IFN-γ response has a hepatic effect to produce liver injury in FHL pathophysiology. Liver resident Kupffer cells and dendritic cells may be resistant to irradiation and may also alter the immune response and affect the inflammatory physiology of FHL [31, 32]. Thus, some of the host effect could be attributed to these cell types. Future work in cell linage specific receptor deleted mice will clarify these issues. The IFN-γ response in the liver alters immune and metabolic pathways corelating with liver injury and is also critical in recruitment of the main immune-mediators of disease, Teff and inflammatory monocytes. This provides novel insight to the mechanism of IFN-γ response in immune pathogenesis of FHL hepatitis.

One of the basic limitations of our model is its inability to differentiate between specific cell populations of liver parenchyma such as hepatocytes, stellate cells, tissue resident immune cells, liver sinusoidal endothelial cells (LSEC) or other non-hematopoietic tissue contributing to FHL pathology. However, significantly increased transcripts of *Stat1*, *Igtp1*, *Irgm1* and *Cxcl9*, all downstream interferon-inducible genes, in *IFNgR*^*+/+*^ livers without a significant contribution of BM IFN-γ-R response, indicate a hepatic intrinsic response is induced during disease. While we cannot completely rule out non-hepatic, non-immune factors and radioresistant hepatic dendritic and Kupffer cells contributing to the liver specific effects of FHL, taken together these data suggest that non-hematopoietic responses to IFN-γ are contributing to liver pathology. Further, elucidation of the various hepatic compartments’ contribution to IFN-γ induced hepatitis in FHL using Cre mediated, lineage specific deletion will be a goal of future work.

We observed significantly higher levels of serum IFN-γ, neutrophilia and weight loss in the mice who lacked IFN-γ-R in both BM and non-immune cells consistent with our description of mice natively deficient in the receptor demonstrating the fidelity of our chimeric system [19]. Platelet count was primarily driven by BM response to IFN-γ but there was a synergistic contribution in ameliorating thrombocytopenia when the non-immune organs could sense IFN-γ via the IFN-γ-R. sIL-2r is a marker for T-cell activation which is elevated in CSS hepatitis from various etiologies [30, 33–35]. Interestingly, we observed that sIL-2r levels were dependent not only on leukocyte response to IFN-γ but also on non-immune organ response in the host synergistically, suggesting a non-immune organ response to IFN-γ may contribute T-cell hyper-activation in FHL. This might be attributed to the liver sinusoidal environment, which regulates both T-cell activation via antigen presentation and recruitment by CXCR3 ligands facilitated by LSEC [36]. However, this could also be due to sIL-2r production by non-immune tissues themselves as was observed clinically by Bode et al. where HLH developed in patients with severe combined immunodeficiency manifesting in an absence of T cells, yet still had elevated sIL-2r [37]. We conclude that while certain IFN-γ induced immunopathology characteristics in FHL are entirely hematopoietic-intrinsic (spleen size), the sensing of IFN-γ by non-immune organs determines certain phenotypic features of FHL such as lymphocyte activation and thrombocytopenia.

Hepatocytes are known to express IFN-γ-R, which assists in innate and type 1 immune response to hepatotropic viruses and other environmental insults to which mammals are exposed [12, 38]. The hepatic response is involved in cell death, recruitment of leukocytes, inhibition of fibrosis and metabolic changes [12, 28, 29, 38]. Prencipe et al. described a hepatic upregulation in transcription of genes involved in the IFN-γ pathway in children suffering from secondary hemophagocytic lymphohistiocytosis (HLH) with hepatitis, supporting the hypothesis that hepatocyte IFN-γ response could contribute to liver injury in HLH [3]. Using our compartmentalization of immune and non-immune responses in the mouse model of FHL2, we were able to demonstrate that the non-hematopoietic IFN-γ response contributes to liver injury in an independent manner from the leukocyte response. The elevation of ALT, hepatomegaly and histological findings of endothelial injury, which were all driven by the non-immune IFN-γ-R phenotype, indicate its response is critical to pathogenesis in FHL hepatitis and are observed in children suffering from FHL [39, 40].

We found that there are IFN-γ-R effects on transcription that were non-hematopoietic mediated resulting in altered hepatic metabolic response as part of immunopathology. These included GST and the oxidoreductases in the CYP450 metabolic pathways, specifically in the cyp2 iso-enzymes. Transcription is modified in various inflammatory environments such as infection, cancer and autoimmune disorders. The main determinants of CYP450 expression are cytokines, particularly IFN-γ, interleukin (IL)-1, IL-6 and tumor necrosis factor α (TNF-α) which modify expression of various isoforms of CYP450 family depending on the inflammatory milieu [41]. Schuck et al. showed in a murine model of nonalcoholic fatty liver disease (NAFLD) that part of the inflammatory pathogenesis in NAFLD is mediated by suppression of activity of various isoforms of the CYP2 family [42]. Similarly, we show that hepatic GST enzymatic activity is suppressed by non-hematopoietic IFN-γ response yet increases in transcription may be compensatory to reduce oxidative stress as part of cytolytic effect of the cytokine. Reduction of oxidative stress is known to enhance type 1 and 2 IFN response against hepatitis C in both animal models and humans [43, 44]. Our findings showing IFN-γ response results in increased transcription of genes involved in the GST pathway but decrease in enzymatic activity, suggests that part of the IFN-γ hepatic response is compensatory to reduce cellular oxidative stress in hepatocytes as has been described in infectious hepatitis [44].

The anti-fibrotic effects of IFN-γ in viral hepatitis have been extensively described in humans and animal models [45–49]. Weng et al. showed a significant decrease in hepatic fibrosis scores of patients with hepatitis B treated with IFN-γ compared to placebo [48]. The same group showed that IFN-γ works as an antagonist to tumor growth factor-β effects in hepatic stellate cells (HSC) resulting in reduction of ECM production [49]. Work in animal models of toxin and infectious fibrosis describe the effects of IFN-γ in reduction of ECM production by direct effect on metabolic pathways in HSC or reduction of number of HSC by Natural Killer cells [38, 46, 47]. In our model the main pathways enriched in IFN-γ-deficient hepatic response as compared to sufficient livers were related to ECM production. This suggests that the protective role IFN-γ has against infectious or toxic or infectious causes of liver injury, may alternatively result in liver dysfunction when produced in a dysregulated manner such as in FHL.

**Fig 6:**
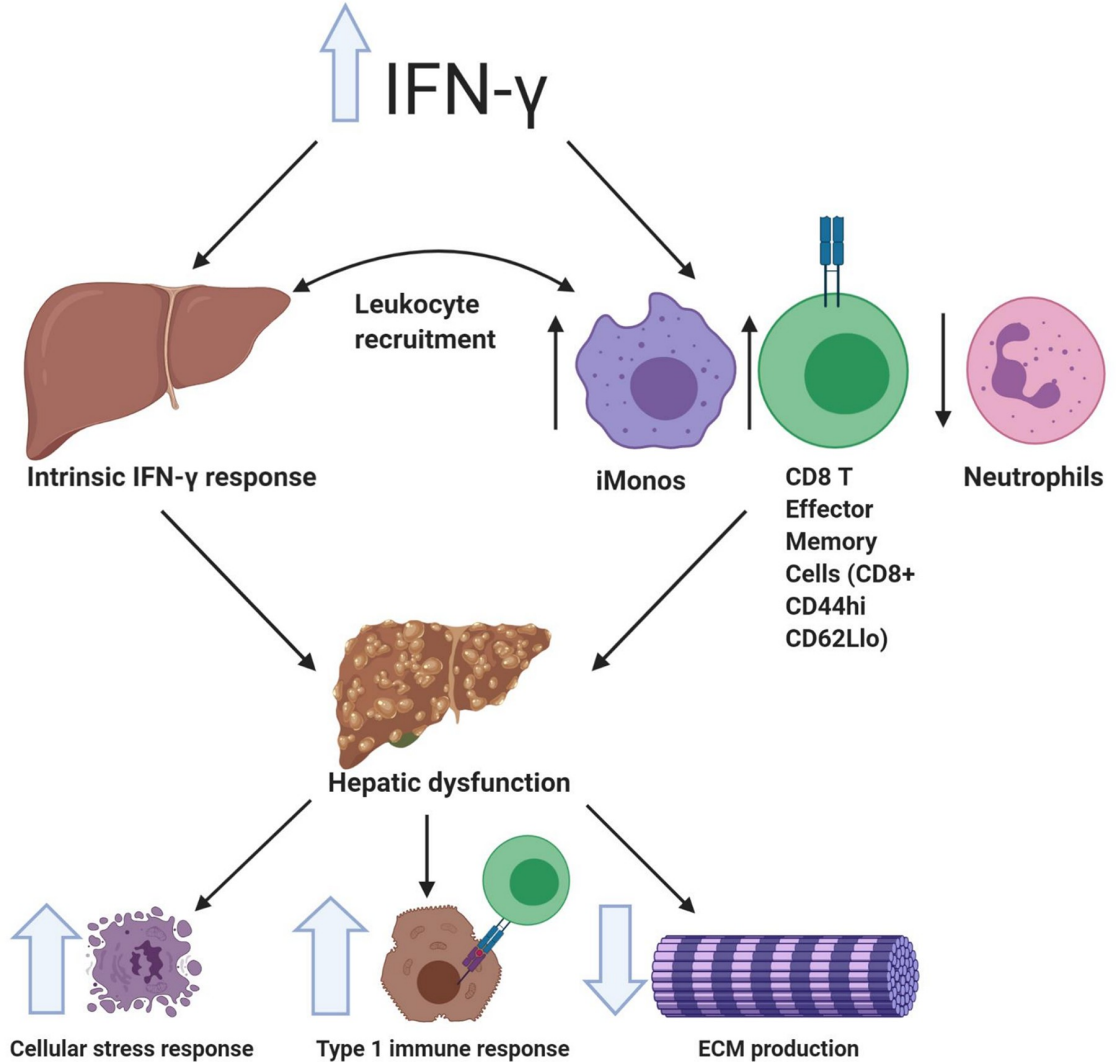
Mechanistic depiction of liver injury in Familial Hemophagocytic Lymphohistiocytosis (FHL) physiology. Elevation of IFN-γ produces liver injury via non-hematopoietic response, which contributes to inflammatory milieu predominated by CTLs, primarily T Effector Cells (CD8+ CD44hi CD62Llo) and inflammatory monocytes (iMonos). Direct effect of IFN-γ on the liver results in increased transcription of type 1 and innate immune response as well as CYP450 metabolism through glutathione transferase and decrease in production of ECM. Created with BioRender.com.

The effects of hepatic response to IFN-γ in recruitment of leukocytes is of great importance as we learn more about T-cell mediated hepatic injury in CSS. Prencipe et al. showed recently that there is hepatic upregulation in *Cxcl9* gene transcripts in a murine model of MAS, as part of the IFN-γ response, similarly to what the group described in human livers [3, 5]. In our current study, we were able to establish this transcriptional response is dependent on the non-immune hepatic IFN-γ-R. By compartmentalization of the IFN-γ response, we describe the pathophysiological effect of this cytokine on hepatic recruitment of cellular mediators of disease and its effect on the inflammatory milieu. The increase in hepatic recruitment of CD8+ T cells and inflammatory monocytes, was driven by non-hematopoietic IFN-γ-R genotype independently from the BM response and correlates to the increase in *Cxcl9* gene transcripts in this group. This work provides evidence that tissue injury by direct cytokine toxicity/response may contribute to FHL inflammation in addition to IFN-γ effects on immune cells. In addition, our findings provide evidence of the important role of the liver, as an immunomodulatory organ, in recruitment of cells critical to the immunopathology of CSS and the interplay between these two compartments (Fig 6).

In summary, our current study demonstrates that non-hematopoietic response to IFN-γ is critical for pathogenesis of FHL hepatitis independent of the immune response. Absence of hepatic IFN-γ response results in amelioration of hepatic dysfunction, endotheliitis, ECM disruption as well as recruitment and activation of immune main mediators of FHL hepatitis and suggest IFN-γ blockade may be viable hepatoprotective strategy in FHL and other CSS.

## Supporting information

S1 Fig Hepatic expression of canonical downstream genes increases in response to IFN-γ. Liver mRNA transcripts of *Cxcl9* (A),*Stat1* (B), *Igtp1* (C) and *Irgm1* (D) were compared between 4 chimera groups using 2 way ANOVA to assess variability between host and BM *IFNgR* genotypes. P-values are denoted in box embedded in the graphs, medians are depicted in the horizontal line. Symbols denote individual mice. AU, arbitrary units.
(TIFF)

S2 Fig Liver gating strategy. Example of gating strategy for intrahepatic leukocytes. Panel A was used to identify B cell (Live, B220+, CD90.2-), T-cell populations (Live, B220-, CD90.2+, CD4+ or CD8+), neutrophils (Live, B220-, CD90.2-, Ly6G+Cd11b+) and inflammatory monocytes (Live, B220-, CD90.2-, Ly6G-, CD11b+, Ly6c+) (A). Panel B was used to identify NK cells (Live, NK1.1+,CD90.2-), NKT cells (Live, NK1.1, CD90.2) and CD8 subpopulations: naïve T cells (Live, NK1.1-, CD90.2+, CD8+, CD44+, CD62L+), T-effector cells (Live, NK1.1-, CD90.2+, CD8+, CD44+, CD62L-) and T-central memory cells (Live, NK1.1-, CD90.2+, CD8+, CD44-, CD62L+).
(TIFF)

S3 Fig Spleen gating strategy. Example of gating strategy for splenocytes. Panel used to identify B cell (Live, B220+, CD90.2-), T-cell populations (Live, B220-, CD90.2+, CD4+ or CD8+) and CD8 subpopulations: naïve T cells (Live, NK1.1-, CD90.2+, CD8+, CD44+, CD62L+), T-effector cells (Live, NK1.1-, CD90.2+, CD8+, CD44+, CD62L-) and T-central memory cells (Live, NK1.1-, CD90.2+, CD8+, CD44-, CD62L+).
(TIFF)

S4 Fig Mobilization of B-cells, Naïve C8+ cells from lymphoid tissue to periphery requires both lymphocyte intrinsic and host response to IFN-γ. Peripheral neutrophils (A), lymphocytes (B) and monocytes (C) counts were compared between 4 chimera groups (n≥5 mice per group) using 2 way ANOVA to assess variability between host and BM *IFNgR* genotypes. Blue represents *IFNgR+/+* and red *IFNgR-/-* in BM (zebra filling) and non-hematopoietic (violin plot boarder). Splenic B-cells (D), Naïve T cell (CD8+ CD44^lo^ CD62L^hi^) (E) and T effector Cell (CD8+ CD44^hi^ CD62L^lo^) (F) counts were compared between 4 chimera groups (n≥15 mice per group) using 2 way ANOVA to assess variability between host and BM *IFNgR* genotypes. P-values are denoted in box embedded in the graphs, medians and quartiles are depicted in the dashed and dotted lines respectively.
(TIF)

S5 Fig Non-hematopoietic IFN-γ response results in decrease expression of hepatic CXCR3 on T lymphocytes. Quantitative assessment of CXCR3+ expression by mean fluorescence intensity (MFI) in T cell population (B220-, CD90.2+)- (A) CD8+ Teff (B220-, CD90.2+, CD8+ CD44hi CD62Llo), (B) CD4+ cells (B220-, CD90.2+, CD4+,CD8-), and (C) inflammatory monocytes (iMonos) (B220-, CD90.2-, Ly6G-, CD11b+, Ly6C+) (D) Absolute numbers of CXCR3+ iMonos in liver parenchyma. (E) Representative CXCR3+ histogram showing decreased expression of surface CXCR3 in Teff population in mice with IFNgR+/+ liver compared to mice with livers deficient in IFNgR. All data was analyzed using 2-way ANOVA with p values denoted in box embedded in the graphs, medians are depicted in the horizontal line. Symbols denote individual mice.
(TIFF)

S6 Fig Liver flow gating strategy panel A. Panel was used to determine CXCR3+ expression. B cells (Live, B220+, CD90.2-) were used as negative control. T-cell populations (Live, B220-, CD90.2+, CD4+ or CD8+), neutrophils (Live, B220-, CD90.2-, Ly6G+Cd11b+) and inflammatory monocytes (Live, B220-, CD90.2-, Ly6G-, CD11b+, Ly6c+) were gated to assess CXCR3+ cells and then MFI to determine expression of receptor.
(TIFF)

S7 Fig Liver flow gating strategy Panel B. Panel was used to determine CXCR3+ expression. B cells (Live, B220+, CD90.2-) were used as negative control. CD8 subpopulations: naïve T cells (Live, CD90.2+, CD8+, CD44+, CD62L+), T-effector cells (Live, CD90.2+, CD8+, CD44+, CD62L-) and T-central memory cells (Live, CD90.2+, CD8+, CD44-, CD62L+) were gated to assess CXCR3+ cells and then MFI to determine expression of receptor.
(TIFF)

S8 Fig Liver Glutathione S-Transferase (GST) activity is decreased by IFNgR signaling. Quantitative assessment of hepatic GST activity using fluorescent activity assay (Thermo Scientific™) showing decreased activity in livers responsive to IFNgR signaling. All data was analyzed using 2-way ANOVA with p values denoted in box embedded in the graphs, medians are depicted in the horizontal line. Symbols denote individual mice.
(TIFF)

S1 Table List of antibodies used. (DOCX)

S2 Table Genes with increased expression in IFNγ-R+/+ livers. (DOCX)

S3 Table Genes with decreased expression in IFNγ-R+/+ livers. (DOCX)

## Abbreviations

CSS: Cytokine Storm Syndromes
FHL: familial hemophagocytic lymphohistiocytosis
MAS: macrophage activation syndrome
IFN-γ: interferon gamma
IFNγ-R: interferon gamma receptor
Teff: CD8+ CD44^hi^ CD62L^lo^ effector T lymphocytes
BM: bone marrow
LCMV: Lymphocytic Choriomeningitis Virus
ALT: alanine aminotransferase
AST: aspartate aminotransferase
CBC: complete blood count
sIL-2r: soluble interleukin-2 receptor
H&E: hematoxylin and eosin
mRNAseq: messenger RNA sequencing
CPM: count per million
GO: gene ontology
DAVID: Database for Annotation, Visualization and integrated Discovery
GEO: Gene Expression Omnibus
PBS: phosphate buffered saline
GST: Glutathione S-Transferase
WBC: white blood cell count
RBC: red blood cell count
CXCL: chemokine ligand
IGTP: interferon gamma induced GTPase
IRGM: immunity-related GTPase family M member
STAT: signal transducer and activator of transcription
CXCR: chemokine receptor
MLKL: Mixed lineage kinase domain like pseudokinase
CYP450: cytochrome P-450
ECM: extra cellular matrix
LSEC: Liver sinusoidal endothelial cells
HLH: hemophagocytic Lymphohistiocytosis
TNF-α: Tumor necrosis factor α
NAFLD: nonalcoholic fatty liver disease
HSC: hepatic stellate cells
