# Supplementary figures and images for: Familial hemophagocytic lymphohistiocytosis hepatitis is mediated by IFN-γ in a predominantly hepatic-intrinsic manner
Source: PLoS One. 2022 Jun 7;17(6):e0269553. doi: 10.1371/journal.pone.0269553 (PMC9173616; doi:10.1371/journal.pone.0269553)

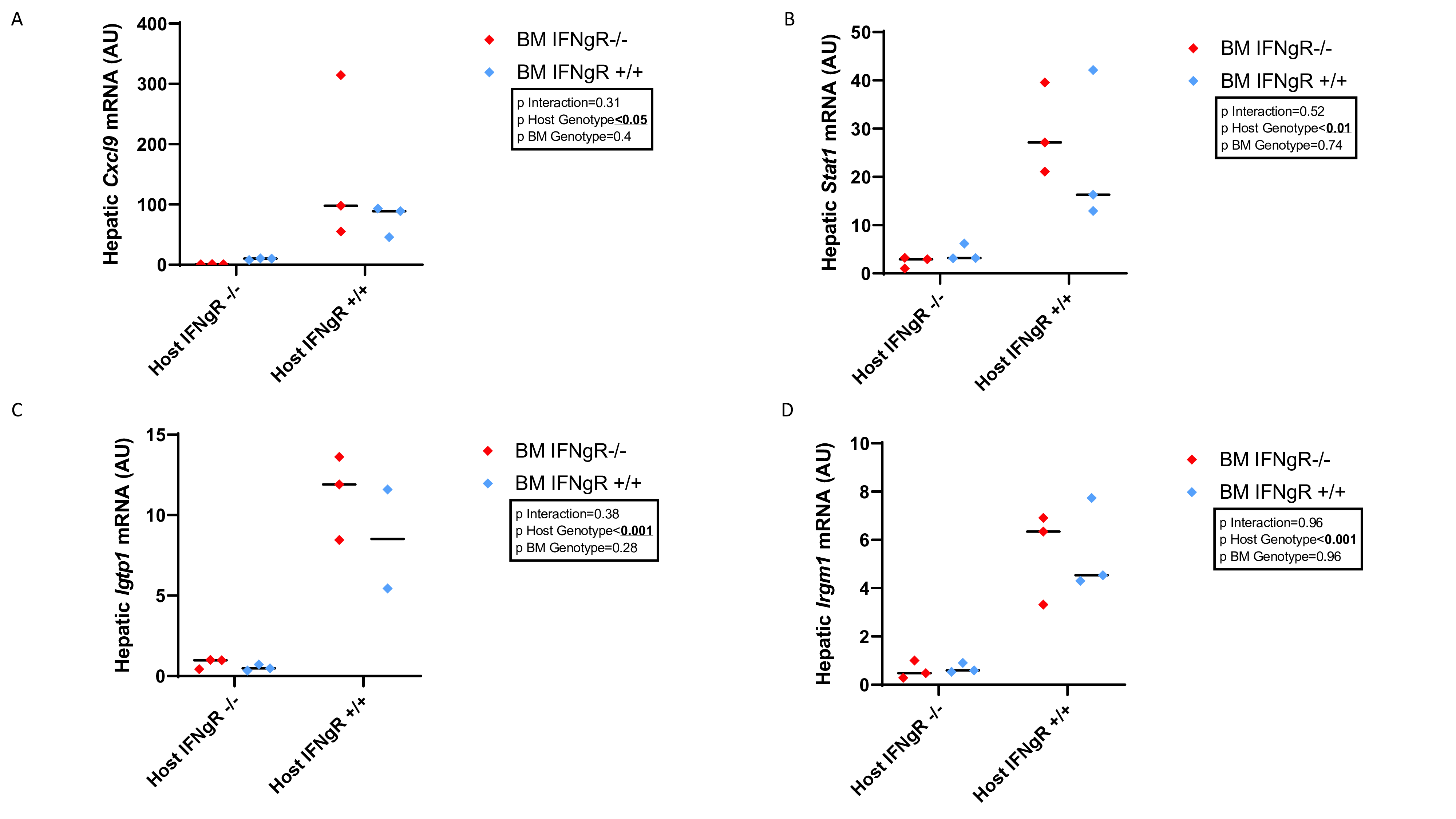

Supplement: S1 Fig — Liver mRNA transcripts of Cxcl9 (A),Stat1 (B), Igtp1 (C) and Irgm1 (D) were compared between 4 chimera groups using 2 way ANOVA to assess variability between host and BM IFNgR genotypes. P-values are denoted in box embedded in the graphs, medians are depicted in the horizontal line. Symbols denote individual mice. AU, arbitrary units. (TIFF) [file pone.0269553.s001.tiff]

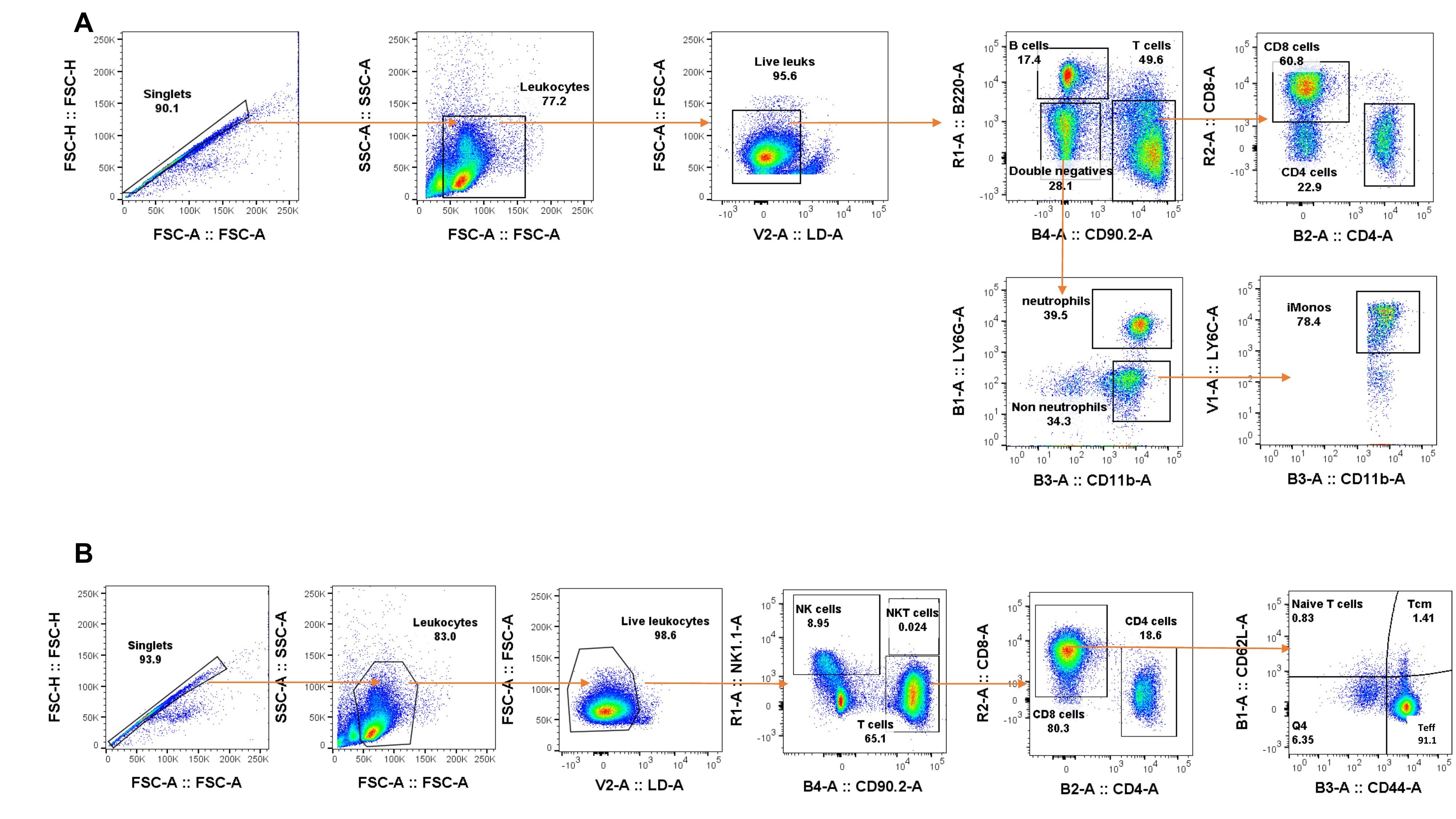

Supplement: S2 Fig — Example of gating strategy for intrahepatic leukocytes. Panel A was used to identify B cell (Live, B220+, CD90.2-), T-cell populations (Live, B220-, CD90.2+, CD4+ or CD8+), neutrophils (Live, B220-, CD90.2-, Ly6G+Cd11b+) and inflammatory monocytes (Live, B220-, CD90.2-, Ly6G-, CD11b+, Ly6c+) (A). Panel B was used to identify NK cells (Live, NK1.1+,CD90.2-), NKT cells (Live, NK1.1, CD90.2) and CD8 subpopulations: naïve T cells (Live, NK1.1-, CD90.2+, CD8+, CD44+, CD62L+), T-effector cells (Live, NK1.1-, CD90.2+, CD8+, CD44+, CD62L-) and T-central memory cells (Live, NK1.1-, CD90.2+, CD8+, CD44-, CD62L+). (TIFF) [file pone.0269553.s002.tiff]

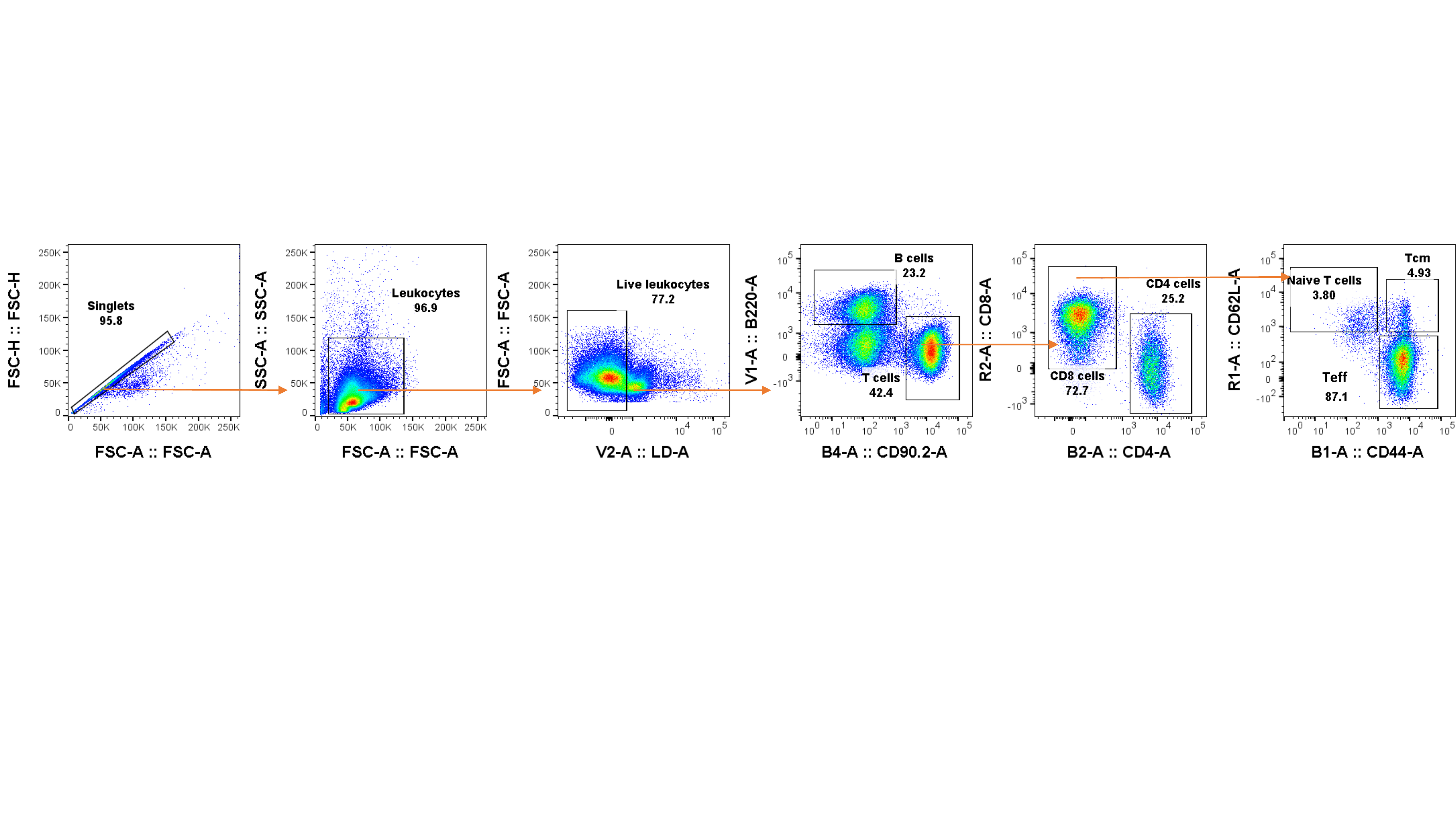

Supplement: S3 Fig — Example of gating strategy for splenocytes. Panel used to identify B cell (Live, B220+, CD90.2-), T-cell populations (Live, B220-, CD90.2+, CD4+ or CD8+) and CD8 subpopulations: naïve T cells (Live, NK1.1-, CD90.2+, CD8+, CD44+, CD62L+), T-effector cells (Live, NK1.1-, CD90.2+, CD8+, CD44+, CD62L-) and T-central memory cells (Live, NK1.1-, CD90.2+, CD8+, CD44-, CD62L+). (TIFF) [file pone.0269553.s003.tiff]

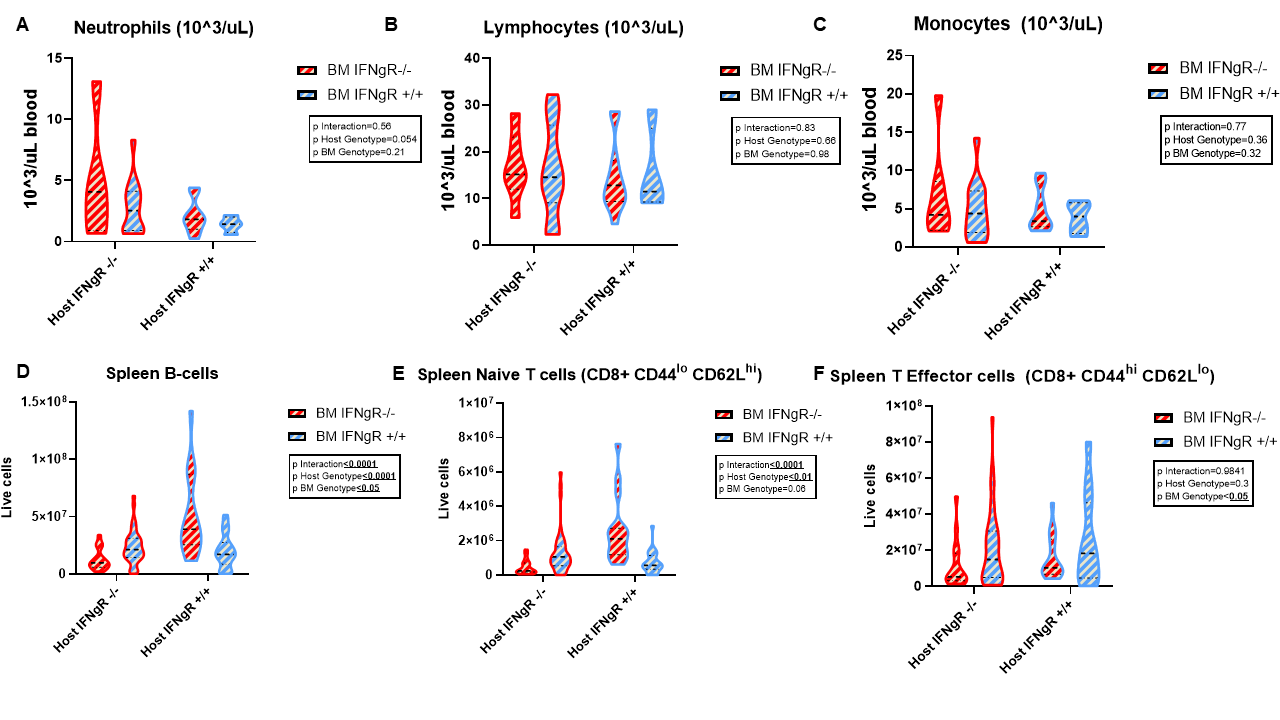

Supplement: S4 Fig — Peripheral neutrophils (A), lymphocytes (B) and monocytes (C) counts were compared between 4 chimera groups (n≥5 mice per group) using 2 way ANOVA to assess variability between host and BM IFNgR genotypes. Blue represents IFNgR+/+ and red IFNgR-/- in BM (zebra filling) and non-hematopoietic (violin plot boarder). Splenic B-cells (D), Naïve T cell (CD8+ CD44lo CD62Lhi) (E) and T effector Cell (CD8+ CD44hi CD62Llo) (F) counts were compared between 4 chimera groups (n≥15 mice per group) using 2 way ANOVA to assess variability between host and BM IFNgR genotypes. P-values are denoted in box embedded in the graphs, medians and quartiles are depicted in the dashed and dotted lines respectively. (TIF) [file pone.0269553.s004.tif]

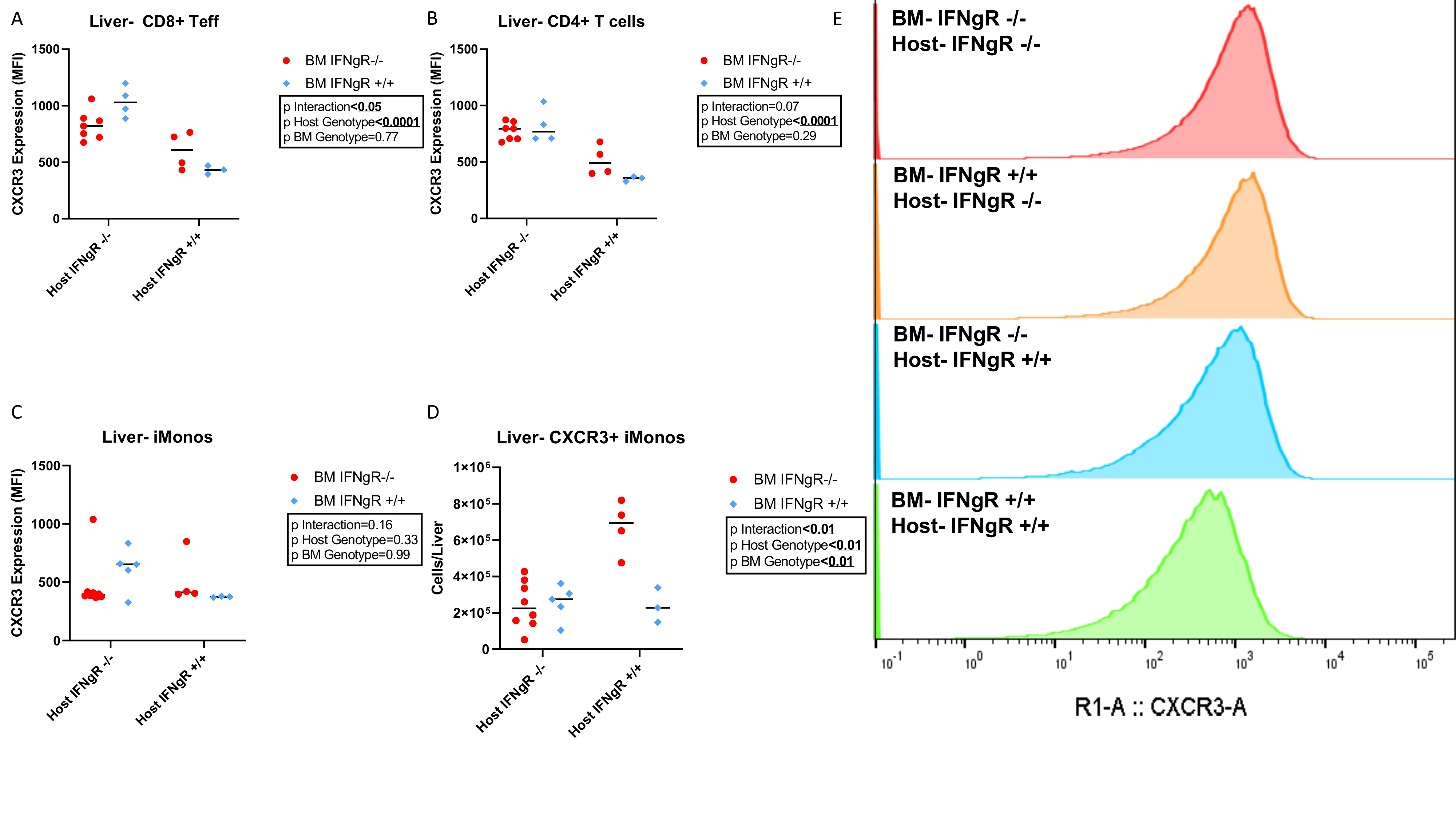

Supplement: S5 Fig — Quantitative assessment of CXCR3+ expression by mean fluorescence intensity (MFI) in T cell population (B220-, CD90.2+)- (A) CD8+ Teff (B220-, CD90.2+, CD8+ CD44hi CD62Llo), (B) CD4+ cells (B220-, CD90.2+, CD4+,CD8-), and (C) inflammatory monocytes (iMonos) (B220-, CD90.2-, Ly6G-, CD11b+, Ly6C+) (D) Absolute numbers of CXCR3+ iMonos in liver parenchyma. (E) Representative CXCR3+ histogram showing decreased expression of surface CXCR3 in Teff population in mice with IFNgR+/+ liver compared to mice with livers deficient in IFNgR. All data was analyzed using 2-way ANOVA with p values denoted in box embedded in the graphs, medians are depicted in the horizontal line. Symbols denote individual mice. (TIFF) [file pone.0269553.s005.tiff]

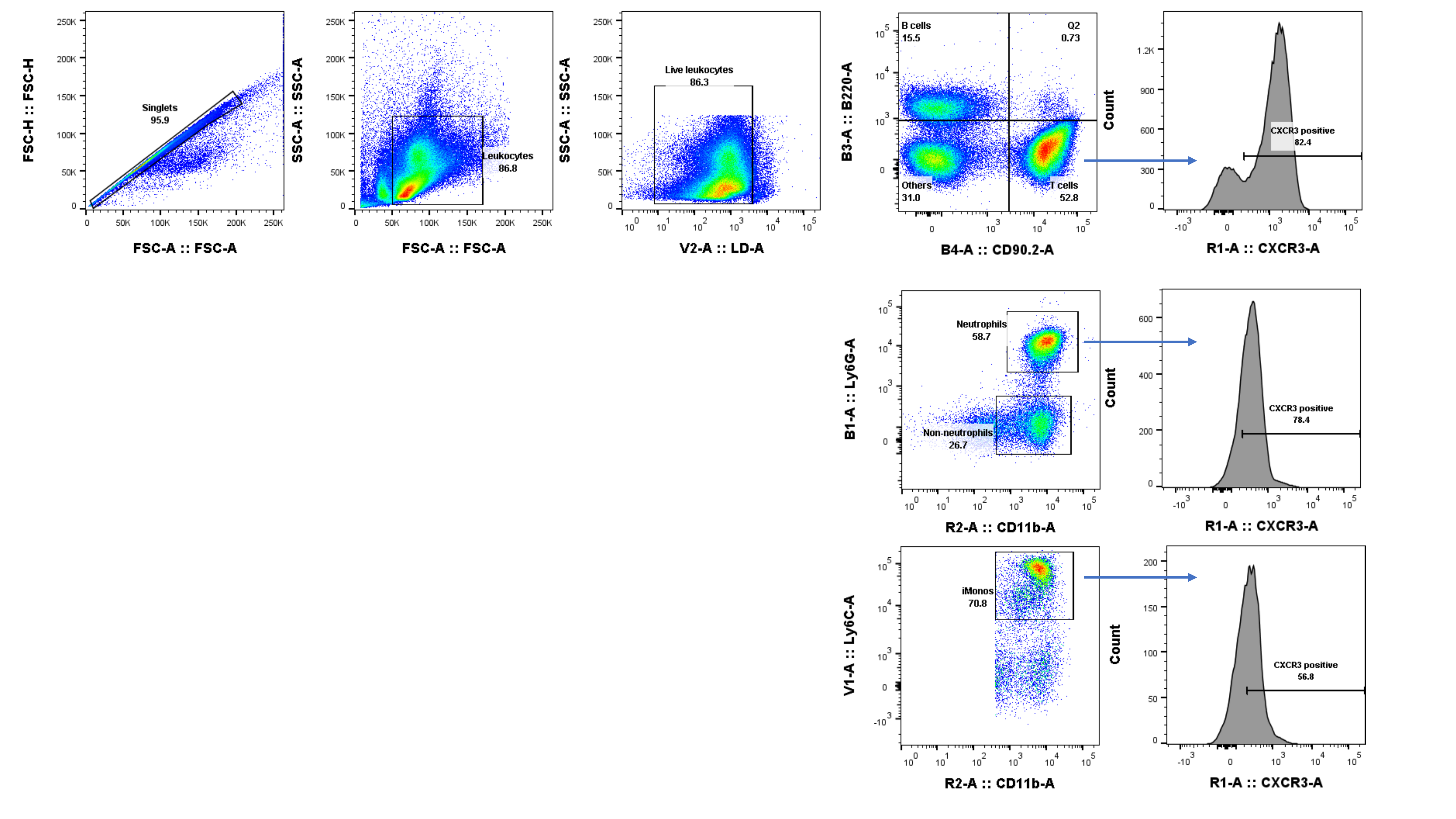

Supplement: S6 Fig — Panel was used to determine CXCR3+ expression. B cells (Live, B220+, CD90.2-) were used as negative control. T-cell populations (Live, B220-, CD90.2+, CD4+ or CD8+), neutrophils (Live, B220-, CD90.2-, Ly6G+Cd11b+) and inflammatory monocytes (Live, B220-, CD90.2-, Ly6G-, CD11b+, Ly6c+) were gated to assess CXCR3+ cells and then MFI to determine expression of receptor. (TIFF) [file pone.0269553.s006.tiff]

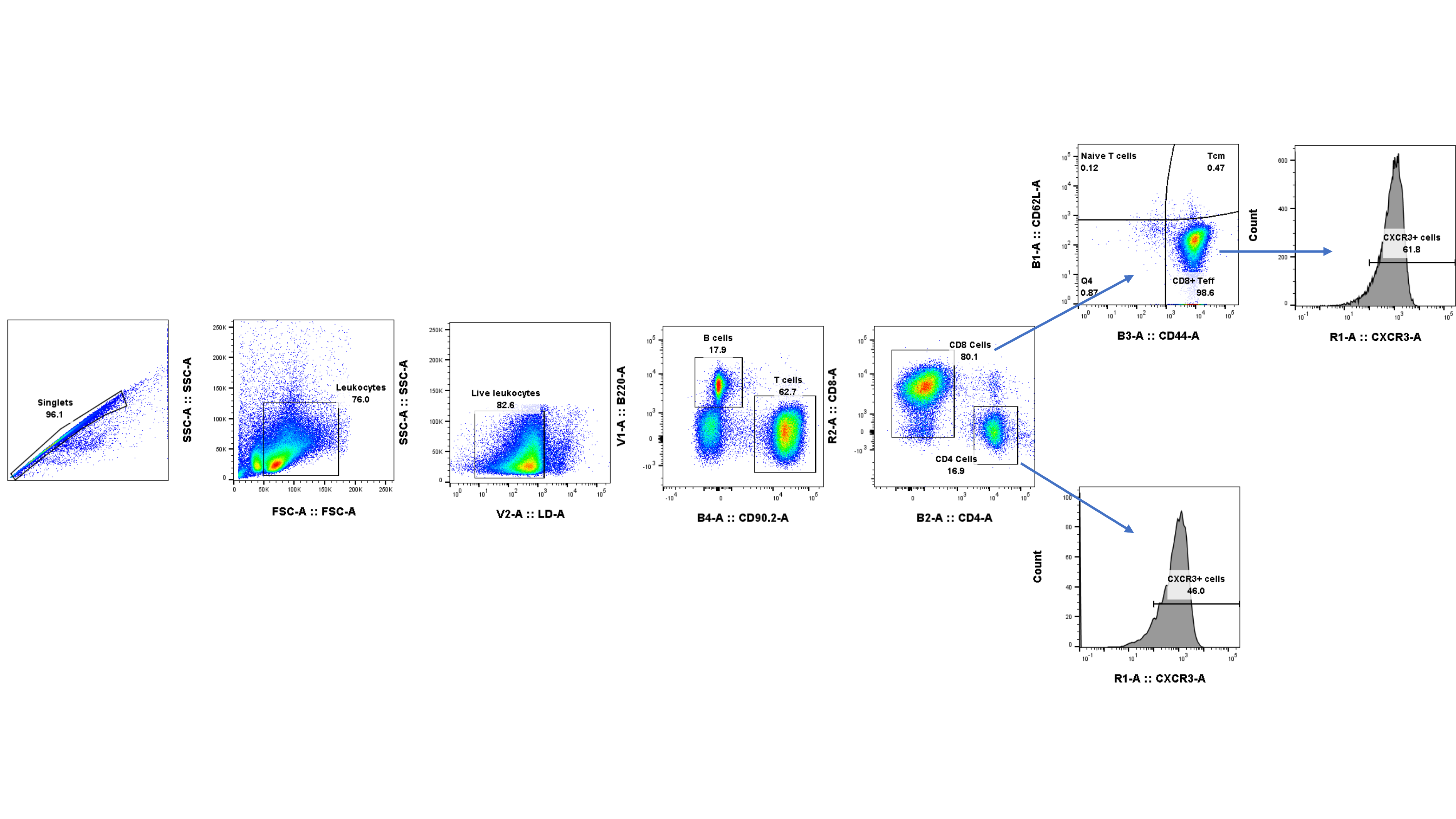

Supplement: S7 Fig — Panel was used to determine CXCR3+ expression. B cells (Live, B220+, CD90.2-) were used as negative control. CD8 subpopulations: naïve T cells (Live, CD90.2+, CD8+, CD44+, CD62L+), T-effector cells (Live, CD90.2+, CD8+, CD44+, CD62L-) and T-central memory cells (Live, CD90.2+, CD8+, CD44-, CD62L+) were gated to assess CXCR3+ cells and then MFI to determine expression of receptor. (TIFF) [file pone.0269553.s007.tiff]

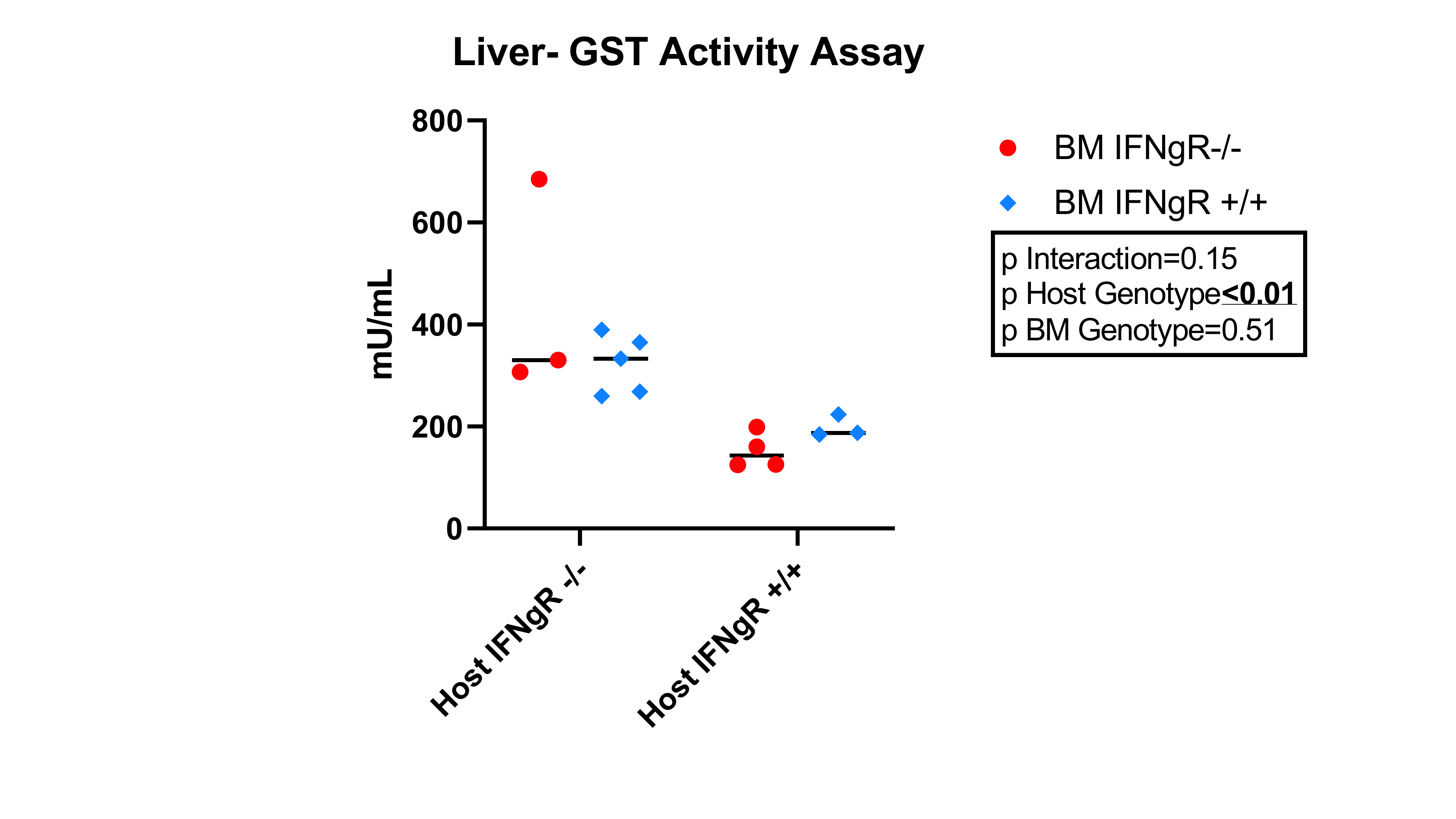

Supplement: S8 Fig — Quantitative assessment of hepatic GST activity using fluorescent activity assay (Thermo Scientific™) showing decreased activity in livers responsive to IFNgR signaling. All data was analyzed using 2-way ANOVA with p values denoted in box embedded in the graphs, medians are depicted in the horizontal line. Symbols denote individual mice. (TIFF) [file pone.0269553.s008.tiff]
