# Supplementary material for: Familial hemophagocytic lymphohistiocytosis hepatitis is mediated by IFN-γ in a predominantly hepatic-intrinsic manner
Source: PLoS One. 2022 Jun 7;17(6):e0269553. doi: 10.1371/journal.pone.0269553 (PMC9173616; doi:10.1371/journal.pone.0269553)
Supplement: S1 Table — (DOCX) [file pone.0269553.s009.docx]

**Supplementary Table 1- List of Antibodies Used**

| **Fluorophore** | **Antibody** | **Manufacturer** |
| --- | --- | --- |
| Pac Blue | Ly6C | BioLegend |
| Pac Blue | B220 | BioLegend |
| Aqua | LIVE/DEAD | Life Technologies |
| FITC | Ly6G | BD Pharmingen |
| FITC | CD44 | BD Pharmingen |
| PE | CD4 | BD Pharmingen |
| PerCP-Cy5.5 | CD11b | BD Pharmingen |
| PerCP-Cy5.5 | CD44 | BioLegend |
| PE-Cy7 | CD90.2 | BD Pharmingen |
| APC | B220 | BioLegend |
| FITC | CD62L | BioLegend |
| APC | NK1.1 | BioLegend |
| APC | CD62L | eBioscience |
| APC-Cy7 | CD8α | BioLegend |
