# Supplementary material for: Familial hemophagocytic lymphohistiocytosis hepatitis is mediated by IFN-γ in a predominantly hepatic-intrinsic manner
Source: PLoS One. 2022 Jun 7;17(6):e0269553. doi: 10.1371/journal.pone.0269553 (PMC9173616; doi:10.1371/journal.pone.0269553)
Supplement: S2 Table — (DOCX) [file pone.0269553.s010.docx]

**Supplementary Table 2- Genes with Increased Expression in IFNγ-R+/+ Livers**

| GeneID | logFC | adj.P.Val |
| --- | --- | --- |
| 1700024P16Rik | -1.007256981 | 0.044062254 |
| 4930519G04Rik | -2.966928397 | 0.023334661 |
| 9030617O03Rik | -0.664629026 | 0.030948281 |
| 9030619P08Rik | -2.188605056 | 0.00247585 |
| Abcg5 | -0.850995752 | 0.032505736 |
| Acmsd | -1.59520752 | 0.027398825 |
| Acot11 | -1.660459018 | 0.005504883 |
| Acot6 | -1.237273098 | 0.032505736 |
| Acox1 | 0.998753058 | 0.025733662 |
| Adam11 | -1.212331123 | 0.038762823 |
| Aifm2 | -1.134834133 | 0.001510907 |
| Akr1b7 | -2.057262863 | 0.027138768 |
| Aldh1a7 | -0.70224122 | 0.04302185 |
| Alox12 | -1.371134193 | 0.0051622 |
| Apol10b | -3.454444752 | 0.028488551 |
| Apol9a | -1.569745167 | 0.012956514 |
| Apol9b | -1.331062092 | 0.047656992 |
| Arhgef16 | -0.950921445 | 0.027054983 |
| BC023105 | -4.255825645 | 0.003817971 |
| Cbr1 | -1.034185082 | 0.017651792 |
| Cd276 | -1.120452904 | 0.035458845 |
| Cd36 | -0.843890096 | 0.024905801 |
| Cdcp1 | -0.945700537 | 0.03944391 |
| Ces2c | -1.924214919 | 0.049233713 |
| Chpt1 | -0.94528971 | 0.021268923 |
| Ciapin1 | -0.616182167 | 0.027398825 |
| Clstn3 | -1.523600884 | 0.021268923 |
| Ctso | -0.788412165 | 0.049560235 |
| Cxcl11 | -1.585834668 | 0.032592237 |
| Cxcl9 | -3.016223663 | 0.002582421 |
| Cyp26a1 | -2.699263913 | 0.015843933 |
| Cyp2a12 | -1.085014699 | 0.046204212 |
| Cyp2g1 | -3.207487135 | 0.025510876 |
| Cyp3a41b | -1.972536384 | 0.019047301 |
| Dclre1a | -1.41371762 | 0.033318621 |
| Ddb2 | -0.880840635 | 0.049217033 |
| Ddit4l | -1.470799986 | 0.0246125 |
| Dennd2d | -0.938802391 | 0.015843933 |
| Eif4e3 | -1.604494656 | 0.0020857 |
| F830016B08Rik | -1.443083375 | 0.019047301 |
| Fabp4 | -0.815977429 | 0.030948281 |
| Fam13a | -1.303854546 | 0.029682719 |
| Fam222a | 0.934940401 | 0.047794508 |
| Fmo5 | -1.191769253 | 0.016842144 |
| Gbp11 | -3.667341885 | 7.18E-04 |
| Gbp2 | -1.762021798 | 0.007113713 |
| Gbp3 | -1.475297703 | 0.015843933 |
| Gbp6 | -4.704900846 | 0.001055448 |
| Gbp7 | -1.700026945 | 0.004516581 |
| Gbp8 | -1.591980382 | 0.049217033 |
| Gbp9 | -2.425846353 | 0.00106867 |
| Gja4 | -1.428418082 | 0.006861225 |
| Gm12216 | -1.350073467 | 0.039550294 |
| Gm12250 | -3.052776832 | 1.51E-04 |
| Gm20547 | -1.405935795 | 0.033318621 |
| Gm28049 | 0.83661851 | 0.025510876 |
| Gm3839 | -2.313591281 | 0.013431031 |
| Gm4841 | -5.288516012 | 2.21E-04 |
| Gsta1 | -3.304457966 | 0.032505736 |
| Gsta2 | -2.734213702 | 0.016842144 |
| Gstm1 | -1.461129775 | 0.036997539 |
| Gstm2 | -1.131280621 | 0.004649823 |
| Gstm3 | -2.844597872 | 0.016842144 |
| Gstm4 | -1.093431279 | 0.007325417 |
| Gstm6 | -0.795864963 | 0.038967392 |
| Gvin1 | -1.572734594 | 0.049217033 |
| H2-D1 | -0.907485505 | 0.025510876 |
| H2-K1 | -0.920900267 | 0.038762823 |
| H2-Q4 | -0.88619748 | 0.011845031 |
| H2-Q6 | -1.014361296 | 0.021260161 |
| H2-T10 | -0.847687203 | 0.034290728 |
| H2-T22 | -1.188427757 | 0.019409708 |
| H2-T23 | -1.130585055 | 0.014098633 |
| Hist1h1c | -1.223566377 | 0.014098633 |
| Hist1h1e | -2.493681908 | 0.033318621 |
| Ifi47 | -1.592348197 | 0.00594091 |
| Igfbp7 | 0.907482755 | 0.004649823 |
| Igtp | -3.369684328 | 1.88E-05 |
| Iigp1 | -2.643829968 | 4.78E-04 |
| Il18bp | -0.963955295 | 0.030948281 |
| Irgm1 | -2.041354256 | 0.001055448 |
| Irgm2 | -2.004027531 | 1.51E-04 |
| Itpk1 | -0.847611031 | 0.048923457 |
| Lrtm1 | -1.110223883 | 0.03191717 |
| Lrtm2 | -2.309410219 | 0.032505736 |
| Ly6a | -1.193715904 | 0.043465649 |
| Meiob | -1.786083965 | 0.034311269 |
| Meis1 | -1.124952717 | 0.043813393 |
| Mfge8 | -0.882628603 | 0.044062254 |
| Micu2 | -0.519321739 | 0.046925043 |
| Mlkl | -1.139593379 | 0.029682719 |
| Mmp7 | -3.887590473 | 0.032377415 |
| Mov10 | -0.821766286 | 0.027398825 |
| Mroh2a | -1.11988848 | 0.025709299 |
| Myh10 | 0.805930881 | 0.023327154 |
| Myo15b | -2.589450231 | 0.034311269 |
| Nqo1 | -1.605723693 | 0.014592925 |
| Parp10 | -0.900453709 | 0.017651792 |
| Parp12 | -1.089650186 | 0.019047301 |
| Pik3c2g | -1.093544516 | 0.027054983 |
| Pir | -0.760472943 | 0.025134654 |
| Pla1a | -1.211273531 | 0.04050375 |
| Pla2g16 | -0.616326387 | 0.049560235 |
| Pld6 | -1.781840378 | 0.032505736 |
| Pls1 | -2.756599018 | 0.012491088 |
| Plscr2 | -0.83171212 | 0.049560235 |
| Ppp1r12b | 0.777523545 | 0.034311269 |
| Prom1 | -3.764754077 | 0.004649823 |
| Psmb10 | -1.05632224 | 0.012491088 |
| Psmb8 | -1.138361607 | 0.012491088 |
| Psmb9 | -1.568086217 | 0.004427178 |
| Psme1 | -1.162663055 | 0.004649823 |
| Psme2 | -0.875506286 | 0.04857061 |
| Raet1d | -2.618442286 | 0.009292162 |
| Raet1e | -2.67839786 | 0.012491088 |
| Rasl10b | -1.966825335 | 0.025301844 |
| Rfx5 | -0.701106229 | 0.043813393 |
| Rgs12 | -2.075042639 | 0.00247585 |
| RHBG | -1.823193016 | 0.005400149 |
| Rhbg | -1.794346636 | 0.012255398 |
| Rmdn3 | -0.807186526 | 0.025510876 |
| Saa2 | -11.62930333 | 0.019445106 |
| Serpinb1a | -0.744415112 | 0.04652396 |
| Sftpd | -2.855942032 | 0.009995741 |
| Sh2d4a | -1.049026827 | 0.032592237 |
| Slc25a17 | -0.807490665 | 0.023327154 |
| Slc35e2 | -0.647292634 | 0.045009771 |
| Slfn9 | -0.716916079 | 0.04171489 |
| Snrpn | -0.765594136 | 0.026073486 |
| Spns2 | -0.651578367 | 0.021260161 |
| Stat1 | -1.959109708 | 0.00262933 |
| Sult1e1 | -3.35380102 | 0.019047301 |
| Tap1 | -1.023723465 | 0.014098633 |
| Tapbpl | -0.878915927 | 0.02316865 |
| Tdo2 | -0.923347404 | 0.029682719 |
| Tgtp1 | -4.118190451 | 1.51E-04 |
| Tgtp2 | -3.48657721 | 6.63E-04 |
| Timp3 | -1.045852363 | 0.024905801 |
| Tmprss4 | -2.716919238 | 0.007325417 |
| Tnfaip8l3 | -1.890451372 | 0.017651792 |
| Tnfsf10 | -1.68651853 | 0.001162321 |
| Trafd1 | -0.552171547 | 0.0456018 |
| Trim12c | -0.897516349 | 0.049217033 |
| Trim21 | -0.831354761 | 0.016842144 |
| Trim5 | -0.803804899 | 0.049560235 |
| Tsku | -2.389599727 | 0.038585674 |
| Tuba8 | -2.777805917 | 0.003352204 |
| Tubb3 | -2.283170946 | 0.010782309 |
| Ubd | -4.949014741 | 0.002739312 |
| Ugt1a9 | -1.531968061 | 0.019047301 |
| Ugt2b37 | -1.193403781 | 0.03623806 |
| Xkr9 | -1.477811383 | 0.001914 |
| Zbp1 | -1.300684094 | 0.045767014 |
| Zfp354a | -0.835001201 | 0.049217033 |
