# Supplementary material for: Familial hemophagocytic lymphohistiocytosis hepatitis is mediated by IFN-γ in a predominantly hepatic-intrinsic manner
Source: PLoS One. 2022 Jun 7;17(6):e0269553. doi: 10.1371/journal.pone.0269553 (PMC9173616; doi:10.1371/journal.pone.0269553)
Supplement: S3 Table — (DOCX) [file pone.0269553.s011.docx]

**Supplementary Table 3- Genes with Decreased Expression in IFNγ-R+/+ Livers**

| GeneID | logFC | adj.P.Val |
| --- | --- | --- |
| Abcc9 | 2.125570066 | 0.016570965 |
| Abi3bp | 2.049527195 | 0.020304841 |
| Ackr1 | 1.780629943 | 0.037785311 |
| Adamdec1 | 1.63935158 | 0.027956898 |
| Adamts13 | 2.385353521 | 0.010288656 |
| Adamtsl2 | 3.511216074 | 0.002098232 |
| Adra2b | 2.141657646 | 0.023334661 |
| Aebp1 | 1.259281432 | 0.043452784 |
| Angptl2 | 2.103370617 | 0.028231423 |
| Ano1 | 1.307595606 | 0.03623806 |
| Arap3 | 1.055977515 | 0.032505191 |
| Bmp5 | 1.935650211 | 0.032505736 |
| Btnl9 | 1.286362809 | 0.049560235 |
| C1qtnf5 | 1.5377911 | 0.019047301 |
| C6 | 2.056754253 | 0.032505736 |
| Cadm3 | 1.831720304 | 0.026424221 |
| Cd207 | 3.415665003 | 0.042852276 |
| Cd5l | 1.342830631 | 0.033784186 |
| Clec1b | 1.088699288 | 0.03623806 |
| Clec4f | 2.487742983 | 6.63E-04 |
| Col14a1 | 2.084984802 | 0.024905801 |
| Col6a1 | 1.861661236 | 0.017651792 |
| Col6a2 | 1.999172377 | 0.023334661 |
| Col6a3 | 2.600935043 | 0.039550294 |
| Colec10 | 2.919624879 | 0.044768132 |
| Colec11 | 2.181169742 | 0.002739312 |
| Cygb | 1.715153282 | 0.023334661 |
| Dcn | 1.747190542 | 0.001162321 |
| Dnaic1 | 2.008624488 | 0.022300247 |
| Dpt | 1.992427322 | 0.004649823 |
| Ear2 | 1.705883605 | 0.005994479 |
| Ecm1 | 1.253018011 | 0.004481008 |
| Enho | 1.284811325 | 0.013936623 |
| Fabp7 | 1.494656962 | 0.034397073 |
| Fam167a | 1.851173144 | 0.034290728 |
| Fcna | 1.395130447 | 0.012491088 |
| Fmod | 1.619154681 | 0.049560235 |
| Folr2 | 1.964890874 | 0.032775931 |
| Gdf10 | 1.505956135 | 0.038762823 |
| Gem | 1.062646385 | 0.038585674 |
| Gm13889 | 1.325414832 | 0.032505736 |
| Gpat2 | 2.136787419 | 0.049749921 |
| Hand2 | 1.961331366 | 0.049560235 |
| Igsf10 | 2.761724313 | 0.049560235 |
| Il34 | 1.439268564 | 0.029740382 |
| Islr | 1.640572499 | 0.043813393 |
| Kcna2 | 2.890549579 | 0.004516581 |
| Leprel2 | 1.366361373 | 0.042862966 |
| Lrat | 1.696357772 | 0.027398825 |
| Ltbp4 | 3.07698536 | 0.001055448 |
| Lum | 1.736169872 | 0.032753487 |
| Mgp | 1.557616252 | 0.027398825 |
| Mlph | 1.7024381 | 0.034311269 |
| Mmp2 | 1.548424956 | 0.04857061 |
| Mup16 | 1.342663013 | 0.016842144 |
| Mup17 | 1.897249416 | 0.019047301 |
| Mup8 | 1.461760619 | 0.015843933 |
| Mustn1 | 1.983237328 | 0.001914 |
| Ngfr | 1.92045131 | 0.007856571 |
| Ntn1 | 2.025151229 | 0.025969511 |
| Pam | 1.229173187 | 0.019047301 |
| Pcolce2 | 2.101139276 | 0.034290728 |
| Pdgfra | 1.670671468 | 0.04302185 |
| Pltp | 1.31151831 | 0.011600868 |
| Plvap | 1.063773084 | 0.018192521 |
| Prune2 | 1.401739392 | 0.034311269 |
| Rbms3 | 1.371014231 | 0.03623806 |
| Reln | 3.18697725 | 0.025134654 |
| Rgs5 | 1.404117215 | 0.028231423 |
| Rims3 | 1.79051832 | 0.034290728 |
| Slc16a9 | 2.222701668 | 0.011600868 |
| Slc1a3 | 1.589266123 | 0.022006791 |
| Slc22a7 | 1.63169701 | 0.049749921 |
| Sod3 | 1.527562935 | 0.005400149 |
| Stab2 | 1.169903145 | 0.033781204 |
| Syt9 | 2.093307402 | 0.012491088 |
| Tmem26 | 1.606571014 | 0.013936623 |
| Vsig4 | 2.142033527 | 0.003248688 |
| Wfdc1 | 1.794850224 | 0.023334661 |
| Xcr1 | 1.372847284 | 0.017038841 |
| Zbtb7c | 1.501786917 | 0.049560235 |
